# Major Genomic Regions for Wheat Grain Weight as Revealed by QTL Linkage Mapping and Meta-Analysis

**DOI:** 10.3389/fpls.2022.802310

**Published:** 2022-02-10

**Authors:** Yongping Miao, Fanli Jing, Jingfu Ma, Yuan Liu, Peipei Zhang, Tao Chen, Zhuo Che, Delong Yang

**Affiliations:** ^1^State Key Laboratory of Aridland Crop Science, Gansu, China; ^2^College of Life Science and Technology, Gansu Agricultural University, Gansu, China; ^3^Plant Seed Master Station of Gansu Province, Gansu, China

**Keywords:** wheat, thousand grain weight, quantitative trait loci, meta-analysis, candidate genes

## Abstract

Grain weight is a key determinant for grain yield potential in wheat, which is highly governed by a type of quantitative genetic basis. The identification of major quantitative trait locus (QTL) and functional genes are urgently required for molecular improvements in wheat grain yield. In this study, major genomic regions and putative candidate genes for thousand grain weight (TGW) were revealed by integrative approaches with QTL linkage mapping, meta-analysis and transcriptome evaluation. Forty-five TGW QTLs were detected using a set of recombinant inbred lines, explaining 1.76-12.87% of the phenotypic variation. Of these, ten stable QTLs were identified across more than four environments. Meta-QTL (MQTL) analysis were performed on 394 initial TGW QTLs available from previous studies and the present study, where 274 loci were finally refined into 67 MQTLs. The average confidence interval of these MQTLs was 3.73-fold less than that of initial QTLs. A total of 134 putative candidate genes were mined within MQTL regions by combined analysis of transcriptomic and omics data. Some key putative candidate genes similar to those reported early for grain development and grain weight formation were further discussed. This finding will provide a better understanding of the genetic determinants of TGW and will be useful for marker-assisted selection of high yield in wheat breeding.

## Introduction

Wheat (*Triticum aestivum* L.) is one of the leading cereal crops and is vital for global food and nutrition security, providing approximately 20% of total calories and proteins for more than 35% of the human population (FAO)^1^. However, the sustainable production of wheat will be confronted with great challenges in the future, owing to ever-growing populations, extreme climate changes and arable land reductions (Curtis and Halford, 2014). It has been estimated that wheat yield must grow at least 2.4% per year to meet food demands in the next 30 years (Ray et al., 2013). In this context, the genetic improvement in grain yield potential is urgently required to achieve future increases in wheat productivity. Grain yield is a complex quantitative trait determined by three components, thousand grain weight (TGW), grain number per spike, and reproductive tiller number (Duan et al., 2020). Among them, TGW is an important trait due to its phenotypic stability with moderate to high heritabilities of 0.6-0.8, and thus serves as a practical selection criterion for increasing grain yield in the wheat breeding process (Wang L. et al., 2012; Avni et al., 2018; Duan et al., 2020). For example, based on the linear regression analysis applied to more than 1850 Chinese wheat varieties released since the 1920s, the average TGW increased from 30.16 g in the 1920s to 38.43 g in the 2010s. The corresponding grain yield increased from 2.01 to 6.58 t ha^–1^, where selection for higher TGW showed a significant contribution to yield improvement (Qin et al., 2015).

Thousand grain weight is a complex quantitative trait governed by polygenes and significantly interacted with environmental factors (Wang L. et al., 2012; Avni et al., 2018; Duan et al., 2020). It is essential to identify major TGW quantitative trait loci (QTL) and further exploit elite genes in the genetic improvement of modern wheat breeding programs. In the last two decades, a large number of QTLs underlying TGW have been successfully identified by traditional bi-parental linkage mapping (Cheng et al., 2015; Hu et al., 2015; Krishnappa et al., 2017; Kumari et al., 2018; Xin et al., 2020; Qu et al., 2021) and genome-wide association approaches (Mir et al., 2012; Yang et al., 2020; Gao et al., 2021). These loci provide a great convenience for revealing the genetic basis of wheat TGW formation. However, the discovery of major and robust QTLs with closely associated markers as a high potential for developing new varieties by the marker-assisted selection (MAS) remains a challenge (Kumar et al., 2020). Most of the reported QTLs showed minor effects and were located in larger QTL intervals, and their expressions were significantly affected by genetic backgrounds and environments (Acuña-Galindo et al., 2015; Kumar et al., 2020; Liu Y. et al., 2020). In addition, some independent or co-localized QTLs did not always have similar loci in different studies, which was usually verified by comparing the flanking markers or comparing with a reference map when co-localized QTLs had a large confidence interval (CI) and might not be identical (Semagn et al., 2013).

As an alternative method of QTL mapping, meta-QTL (MQTL) analysis provides an effective strategy for validating consistent QTLs by integrating independent QTLs from different trials on a consensus or reference map (Goffinet and Gerber, 2000; Semagn et al., 2013; Acuña-Galindo et al., 2015; Soriano and Alvaro, 2019; Kumar et al., 2020; Liu H. et al., 2020; Liu Y. et al., 2020; Yang et al., 2021). The statistical power of MQTL analysis can refine genomic regions that are most frequently involved in trait variation and narrow down the QTL confidence intervals (CI) (Goffinet and Gerber, 2000; Soriano and Alvaro, 2019). Consequently, the integrated MQTLs are not affected by the genetic background, population type, and planting environment in the previous independent experiments. It is facilitated to discover more reliable and consistent QTLs/markers and further identify candidate genes for map-based cloning and MAS in breeding application (Goffinet and Gerber, 2000; Semagn et al., 2013; Acuña-Galindo et al., 2015; Soriano and Alvaro, 2019; Kumar et al., 2020; Liu H. et al., 2020; Liu Y. et al., 2020; Yang et al., 2021). Genome-wide MQTL analysis has been successfully applied in wheat genetic breeding. It has also achieved good insights into the QTL-integration of various quantitative traits in wheat, such as yield-related traits (Acuña-Galindo et al., 2015; Tyagi et al., 2015; Quraishi et al., 2017; Avni et al., 2018; Kumar et al., 2020; Liu H. et al., 2020; Yang et al., 2021), grain quality traits (Quraishi et al., 2017; Soriano et al., 2021), root-related traits (Soriano and Alvaro, 2019), flowering date (Hanocq et al., 2007), pre-harvest sprouting tolerance (Tyagi and Gupta, 2012), drought and heat tolerance (Acuña-Galindo et al., 2015; Kumar et al., 2020; Soriano et al., 2021), disease resistance (Soriano and Royo, 2015; Cai et al., 2019; Liu Y. et al., 2020). The MQTL analysis surveyed relevant QTL studies and refined the CIs of QTLs or QTL clusters to mine more reliable QTLs. However, most of those studies did not investigate the candidate genes behind the MQTL, due to the limitations of the wheat genome sequence. The step-change made recently in wheat genomes is the release of hexaploid wheat Chinese spring high-quality reference genome (International Wheat Genome Sequencing Consortium [IWGSC] et al., 2018). In the same way, a large number of wheat transcriptomic data has been made available in a user-friendly platform (Borrill et al., 2016; Ramírez-González et al., 2018). All these genomic resources present an unprecedented opportunity to unveil the genetic architecture and to mine candidate genes of grain yield and its components in wheat at the levels of physical map and functional genes (Liu H. et al., 2020; Yang et al., 2021). For instance, Kumar et al. (2020) conducted an MQTL analysis of drought tolerance in wheat and identified 13 MQTLs, four of which related to yield and yield-related traits. Interestingly, MQTL4 was a major MQTL with potential for MAS breeding, and three major candidate genes were identified within the MQTL. Likewise, 86 MQTLs were revealed from 381 QTLs for yield and its components, and finally 18 candidate genes or gene clusters were validated by Liu H. et al. (2020). Based on the large-scale integration of meta-QTL and genome-wide association study, Yang et al. (2021) discovered 76 high-confidence MQTL regions and 237 candidate genes that affected wheat yield and yield-related traits. All these candidate genes as reviewed were classified functionally into five groups by Nadolska-Orczyk et al. (2017), including (1) transcription factors regulating spike development; (2) genes involved in metabolism or signaling of growth regulators; (3) genes determining cell division and proliferation mainly impacting grain size; (4) floral regulators influencing inflorescence architecture and in consequence seed number; and (5) genes involved in carbohydrate metabolism affecting plant architecture and grain yield. In particular, many key genes cloned via a homology-based approach were also confirmed within yield-related MTQTL regions (Quraishi et al., 2017; Kumar et al., 2020; Liu H. et al., 2020; Soriano et al., 2021; Yang et al., 2021), such as *TaVrn1* (Yan et al., 2003), *TaVrn2* (Yan et al., 2004), *TaVrn3* (Yan et al., 2006), *TaPpd* (Beales et al., 2007; Díaz et al., 2012), *TaRht* (Díaz et al., 2012), *TaGSD1* (Zhang et al., 2014), *TaCKX2* (Zhang et al., 2011), *TaGW2* (Yang et al., 2012), *TaTGW6* (Hanif et al., 2015), and *TaSus* (Jiang et al., 2011), etc. This thus suggested that MQTL analysis combined with the wheat reference genome is one of desirable strategies for discovering functional genes underlying grain yield-related traits in wheat (Liu H. et al., 2020; Liu Y. et al., 2020; Yang et al., 2021). However, only a few key genes for TGW in wheat have been isolated by map-based cloning (Xu et al., 2019; Chen et al., 2020; Zhao et al., 2021). The molecular basis of QTL/genes governing TGW is still limited. Owing to a narrow genetic background of the biparental populations analyzed or a lack of tight linkage to functional genes, some markers are not efficiently applied for MAS or molecular breeding design in wheat (Duan et al., 2020).

In this study, major genomic regions and putative candidate genes for wheat grain weight were revealed by QTL linkage mapping and meta-analysis. The objective was to (i) identify QTLs for TGW using a RIL (recombinant inbred line) population under multi-environmental conditions; (ii) conduct a reference-based MQTL analysis of TGW QTL data published in recent years and the present mapping results; (iii) further integrate the MQTL analysis and transcriptome evidence to discover the key genomic regions and essential putative candidate genes governing TGW trait in wheat. This finding will provide a well-understanding of the genetic determinants of TGW and lay a foundation for the identification of the reliable QTLs and the prediction of putative candidate genes in wheat genetic improvement.

## Materials and Methods

### Plant Materials and Field Trials

A set of 120 F_8_-derived recombinant inbred lines (RILs) was developed from the cross between two Chinese winter wheat varieties, Longjian 19 and Q9086 (Yang et al., 2016a,b; Li et al., 2020). The male parent Longjian 19 is an elite drought-tolerant cultivar widely grown in rainfed areas (300-500 mm annual rainfall) in northwestern China. The female parent Q9086 is a high-yielding cultivar suitable for cultivation under sufficient water and high fertility conditions but is prone to premature senescence under terminal drought stress. The two parents differed significantly from TGW and other grain yield components (Hu et al., 2015; He et al., 2020; Li et al., 2020; Zuo et al., 2020). Field trials were carried out at Yuzhong farm station, Gansu, China (35°48′N, 104°18′E, 1860 m ASL) in six years from 2013 to 2018, denoted in turns as E1 to E6, respectively, and at Tongwei farm station, Gansu, China (35°110′N, 105°190′E, 1740 m ASL) in 2017 and 2018, denoted as E7 to E8, respectively. The two growing sites are characterized by a typical arid inland climate of northwest China, where the annual average temperature is about 7.0°C, the annual rainfall is below 400 mm with nearly 60% occurring from July to September, but the annual evaporation capacity is more than 1,500 mm. All progenies and parents were sown in late September and harvested in early July of the following year. Field trials at each site were managed under rainfed conditions with the rainfall from 128 (E3) to 236 mm (E8) in each growing season (Supplementary Figure 1). Before sowing, a total of 180 kg nitrogen (N) ha^–1^, 150 kg P_2_O_5_ ha^–1^, and 75 kg K_2_O ha^–1^ were uniformly applied to the soil surface of the entire experimental site, and all wheat plants were no longer fertilized during the growing periods. Field experimental designs were randomized complete blocks with three replications for each line and parent. Each plot was 1 m long with six rows spaced 20 cm apart. Approximately 60 seeds per row were sown. Field management aspects followed the local practices during wheat production.

### Phenotypic Evaluation and Statistical Analysis

At the grain maturity stage, five plants per plot were randomly sampled in each of the environments. After threshing, grains were air-dried and weighed to obtain TGW. TGW was measured by the SC-G2 kernel testing equipment developed by Wanshen Science and Technology Ltd. (Hangzhou, China). TGW phenotypic values from the eight environments were determined as the mean of each family from three replicates. The calculations of descriptive statistics, correlation analysis, analysis of variance (ANOVA), and the best linear unbiased prediction (BLUP) value for TGW in different environments were performed using SPSS 19.0 software by IBM, Armonk, NY United States. The broad-sense heritability (*h*^2^_B_) for TGW was estimated with the formula proposed by Toker (2004). Here, *h*^2^_B_ = σ*_*g*_*^2^*/(*σ*_*g*_*^2^ + σ*_*ge*_*^2^*/r* + σ*_*e*_*^2^*/re*), where σ*_*g*_*^2^, σ*_*ge*_*^2^ and σ*_*e*_*^2^ were estimates of genotype, genotype × environment interaction (GEI) and residual error variances, respectively, and *e* and *r* were the numbers of environments and replicated per environment, respectively.

### Genetic Map and Quantitative Trait Loci Analysis

The genetic linkage map employed in this study was previously developed using a RIL population (Hu et al., 2015; Yang et al., 2016a,b; He et al., 2020; Li et al., 2020; Zuo et al., 2020). The genetic map consisted of 524 simple sequence repeat (SSR) markers covering 21 chromosomes of wheat. The total length was 2266.7 cM with an average distance of 4.3 cM between adjacent markers. The BIP (Biparental populations) module of the software QTL IciMapping version 4.1 (Li et al., 2010) was utilized to identify QTLs for TGW traits based on phenotypic values from eight single environments and the BLUP dataset. The probability in stepwise regression (PIN) parameter value was set at the level of 0.001 with the scanning step size of 1 cM, and the logarithm of odds (LOD) threshold was set at 2.5 to detect the presence of a significant QTL. The QTL interval on the genetic map was defined as the genetic distance between the two flanking markers of the QTL peak. A QTL detected repeatedly across more than four individual environments was considered as a stable QTL. The locations of individual QTLs were drawn on genetic maps using MapChart 2.32 (Voorrips, 2002).

### Initial Quantitative Trait Locus Projection and Meta-QTL Analysis

The initial QTLs for TGW collected from earlier studies and the present QTL mapping results were integrated to conduct QTL projection and MQTL analysis further. For each study, the necessary information was collected as the type of QTL mapping population (F_2_, DH, RIL and Backcross), size of the mapping population, LOD value, QTL position, flanking or closely linked marker, CI and phenotypic variance explained (PVE) value (Yang et al., 2021). After collection of QTL database, all individual QTLs were projected onto a reference genetic map by BioMercator v4.2.3 (Sosnowski et al., 2012). The reference genetic map from two identified genetic maps (Maccaferri et al., 2015; Soriano and Alvaro, 2019) were integrated as high-density reference maps (Bilgrami et al., 2020). This map contained 14548 markers, including SSR, DArT, SNP and other types of markers. The total length is 4813.72 cM with a range of 155.6 cM to 350.11 cM across the 21 linkage groups. The initial QTL data and related individual genetic maps from earlier studies and the reference genetic map were used as input files to construct a consensus map and to further perform MQTL analysis (Yang et al., 2021). For those QTL lacking flanking markers and Cis, the 95% CI was calculated by Darvasi and Soller (1997) and Guo et al. (2006). Of each equation, CI = 530/(N × R^2^), 163/(N × R^2^) and 287/(N × R^2^) were applied for F_2_/Backcross, RIL and DH population, respectively, where N was the size of the mapping population used for QTL analysis, and R^2^ was the PVE of each initial QTL. The QTLs that could not be localized to the consensus map and those localized outside the consensus map were discarded. MQTL analysis was carried out using BioMercator V4.2.3 (Sosnowski et al., 2012). On each chromosome, MQTL analysis were calculated using the two-step algorithm (Veyrieras et al., 2007). An estimator of model fitting, was used to select the best model for the representing the number of MQTL or “real” QTL by five statistical methods, such as the Akaike Information Criterion (AIC), AIC correction (AICc), AIC3 candidate models (AIC3), Bayesian information criterion (BIC) and average weight of evidence (AWE). The algorithms and statistical procedures in the software were well-described previously (Veyrieras et al., 2007; Sosnowski et al., 2012). As a requirement of the method proposed by Venske et al. (2019), the meta-analysis was performed with chromosomes including as a minimum of 10 projected QTLs. Otherwise, attempts to run analysis when < 10 QTLs were projected returned in error (Venske et al., 2019).

### Mapping of Meta-Quantitative Trait Locus on the Wheat Genome

Putative candidate genes were the genes localized within MQTL regions, which were detected based on the positions of flanking marker regions of the MQTL CIs (or the marker closest to the flanking markers). The flanking markers within target MQTLs were searched by the function of ‘‘Marker information’’ in the Triticeae Multi-omics Center^2^ to determine the physical locations. If the physical locations of flanking markers were not found, the sequences of flanking markers were searched from GrainGenes database^3^ or DArT database^4^. And the most likely physical location was further identified by the Blastn program, based on Chinese Spring RefSeq V1.0 chromosomes in the Triticeae Multi-omics Center (See Text Footnote 2).

### Identification of Putative Candidate Genes

Putative candidate genes within MQTL regions were identified by two following methods. (i) The homology-based candidate gene mining (Yang et al., 2021) was given with the close evolutionary relationship between the genomes of Gramineae species (Gaut, 2002). Homology analysis of wheat with model crop rice could broaden our understanding of wheat genes. Key putative candidate genes within the MQTL region were mined using wheat-rice orthologous comparison strategy. Basic information of all grain weight genes in rice was obtained from the China Rice Data Center^5^. The homologs genes in wheat were found using Triticease-Gene Tribe^6^, based on IWGSC RefSeqv1.1. The genes located within the MQTL region were considered to be important putative candidate genes affecting wheat grain weight (Yang et al., 2021). (ii) When the MQTLs were available, the preferred criteria of MQTL proposed by Venske et al. (2019) were conducted as follows: (1) the MQTL was generated through the projection of at least two overlapping QTLs; (2) the physical interval corresponding to the 95% CI was less than 20 Mb at the Chinese Spring wheat reference genome; (3) the genetic distance was shorter than 1.0 cM. For that, high-confidence genes within each highly refined MQTL were then listed and thereafter called putative candidate genes using the Triticeae Multi-omics Center (See Text Footnote 2), based on IWGSC_v1.1_HC_gene annotated genomic features.

### Expression Analysis of Putative Candidate Genes

The transcriptomic data of multiple tissues in bread wheat var. Chinese Spring from expVIP platform^7^ was obtained to identify the differential expression characteristics of putative candidate genes within the target MQTLs (Borrill et al., 2016). The transcriptomic data included five tissues at different growth stages, such as grain at 2, 14, and 30 days after anthesis (DAA); spike at two nodes detectable, flag leaf and anthesis stages; leaf at the seedling, tillering stages and two DAA; stem at the 1 cm spike, two nodes detectable, and anthesis stages; root at the seedling, three leaf and flag leaf stages. Expression levels of putative candidate genes were evaluated by transcripts per million (TPM) values (See Text Footnote 7) and displayed using the TBtools^8^ of TPM, based on normalized scale method. Gene Ontology (GO) term analysis within MQTL intervals was conducted with the GENEDENOVO cloud platform^9^.

## Results

### Phenotypic Variation and Correlation Analysis

**FIGURE 1:**
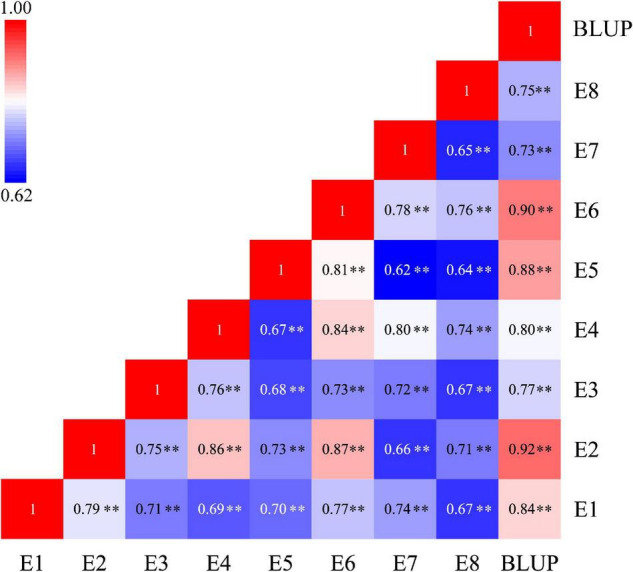
Heatmap depicting the significant correlation of TGW traits under eight tested environments and BLUP analysis. TGW, thousand grain weight; E1-E6, experimental environments at Yuzhong farm station in six years from 2013 to 2018, respectively; E7 and E8, experimental environments at Tongwei farm station in 2017 and 2018, respectively; BLUP, best linear unbiased prediction; ^**^P < 0.01.

The phenotypic values of the RILs and two parents were shown in Table 1. In eight tested environments and BLUP analysis, the parent Q9086 had higher TGW than that of the parent Longjian 19, which differences reached a significant (*P* < 0.05) or very significant level (*P* < 0.01). The mean values of the RILs were intermediate between two parents. The corresponding coefficients of variation ranged from 6.08 to 12.80% in response to different environments. Some progenies had extreme values more than either parent. The absolute values of skewness and kurtosis were less than 1.0. This suggested that TGW traits showed wide phenotypic variability with continuous variation and transgressive segregation in the RILs. The correlation analysis exhibited a very significant and positive correlation among TGW traits in different environments (*P* < 0.01). The correlation coefficients ranged from 0.62^**^ to 0.92^**^, and rainfall for each year was significantly and positively associated with TGW (*r* = 0.54, *P* < 0.05) (Figure 1 and Supplementary Figure 2). The variance component analysis showed that all the variance values in the RILs reached a very significant level (*P* < 0.01), where the phenotypic variation of TGW was highly influenced by the environment, genotype, and GEI (Supplementary Table 1). However, the high value of broad-sense heritability (*h*^2^_B_ = 0.77) indicated that TGW was mainly determined by the genetic factor.

**TABLE 1:** Summary statistics of TGW in the parents and the wheat RIL population under eight environments.

Environments	Parents					*RILs*		
	Longjian 19	Q9086	Mean ± SD	Min	Max	Skewness	Kurtosis	CV (%)
E1	34.76	41.62**	35.86 ± 4.59	25.43	50.46	0.48	0.33	12.80
E2	42.57	47.82**	45.37 ± 4.05	35.03	55.99	–0.24	–0.04	8.93
E3	31.33	36.45**	32.31 ± 3.82	22.87	40.30	–0.35	–0.66	11.81
E4	44.06	47.01*	46.21 ± 2.85	37.77	53.99	0.02	0.24	6.16
E5	35.11	38.39**	36.77 ± 2.51	30.12	43.56	0.11	–0.21	6.83
E6	40.93	45.67**	44.43 ± 2.88	37.91	52.28	0.40	–0.20	6.48
E7	43.77	50.33*	49.71 ± 3.02	42.63	60.50	0.52	0.65	6.08
E8	42.06	46.09*	45.71 ± 3.94	33.27	58.41	0.41	0.57	8.62
BLUP	39.69	41.89*	40.85 ± 3.97	36.45	46.66	0.12	–0.08	9.72

TGW, thousand grain weight; SD, standard deviation; Min, minimum; Max, Maximum; CV, coefficient of variation; BLUP, best linear unbiased prediction. E1-E6, experimental environments at Yuzhong farm station in six years from 2013 to 2018, respectively; E7 and E8, experimental environments at Tongwei farm station in 2017 and 2018, respectively. Field experimental designs under each environment were randomized complete blocks with three replications for each line and parent. The asterisks in the column of “parent Q9086” represent significant differences in phenotypic data between two parents by the F test; *P< 0.05, **P< 0.01.

### Quantitative Trait Locus Mapping for Thousand Grain Weight

**FIGURE 2:**
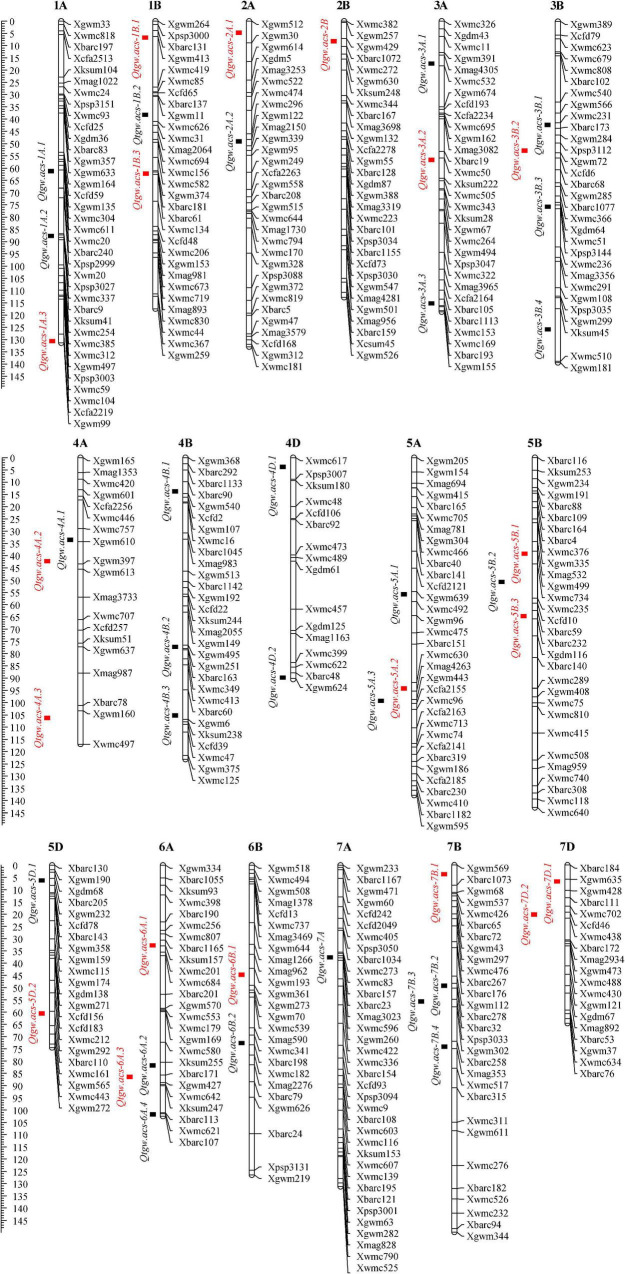
Genetic map with additive QTLs for TGW under eight tested environments and BLUP analysis. The squares represent the locations of QTLs. The black squares are QTLs expressed only in one environment and red squares are expressed repeatedly in at least two environments.

A total of 45 additive QTLs for TGW were detected in eight tested environments and BLUP analysis. These loci were distributed on almost all chromosomes except for 1D, 2D, 3D and 6D and exhibited individual PVE of 1.76-12.87% (Figure 2 and Supplementary Table 2). Of these, 22 QTLs (48.89%) had negative effects with -0.24 to -1.72, indicating favorable allele contribution from the parent Longjian 19. The other 23 loci (51.11%) showed positive effects with 0.37 to 1.50 conferred by favorable alleles from Q9086. This indicated that favorable alleles controlling the TGW trait were almost evenly contributed by the parents. In addition, 25 QTLs (55.56%) were identified in single environments, implying that these QTLs were expressed as an environment-dependent pattern. Most of these loci individually explained lower PVE from 1.76% to 8.79%, and only three loci (*Qtgw.acs-4D.1*, *Qtgw.acs-4D.2* and *Qtgw.acs-5A.1*) had higher PVE from 9.76 to 10.96%. The rest 20 of 45 QTLs (44.44%) were detectable repeatedly across two or more environments and BLUP analysis, indicative of the features of stable expressions. In particular, three stable QTLs (*Qtgw.acs-2B*, *Qtgw.acs-5B.1*, and *Qtgw.acs-5B.3*) were identified across four individual environments, with individual PVE of 6.65-12.23%. Other seven stable QTLs, such as *Qtgw.acs-1A.3*, *Qtgw.acs-1B.1*, *Qtgw.acs-2A.1*, *Qtgw.acs-4A.2*, *Qtgw.acs-6B.1*, *Qtgw.acs-7B.1*, and *Qtgw.acs-7D.1*, were frequently expressed in four to six individual environments and BLUP analysis, accounting for individual PVE of 6.74-12.87%. This suggested that these ten loci were significantly stable QTLs for TGW.

### Characteristics of Initial Quantitative Trait Locus for Thousand Grain Weight

**FIGURE 3:**
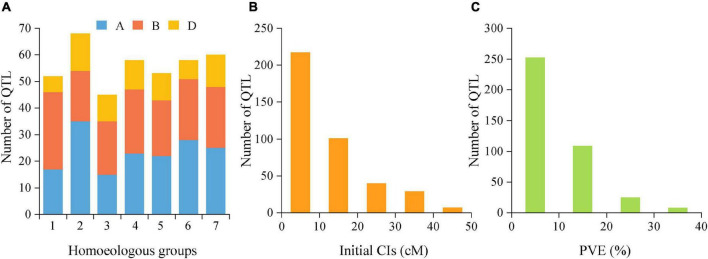
**(A)** Number of QTLs on seven homoeologous groups (1-7) and three sub-genomes **(A,B,D)** from the collected QTL studies. **(B)** supporting intervals estimated from the initial QTLs. **(C)** the individual PVE from QTLs. PVE, phenotypic variance explained.

The reported QTLs for TGW were collected from 45 earlier studies published from 2003 to 2020 that were employed in 39 bi-parental mapping populations, including 22 RIL populations, ten double haploids (DH) populations, five F_2_ populations and two backcross populations (Supplementary Table 3). By integrating these earlier reported 349 QTLs and 45 QTLs detected in this study, a total of 394 initial QTLs for TGW were employed for meta-analysis. These loci were distributed on all 21 chromosomes belonging to seven homoeologous groups (1-7) and three sub-genomes (A, B, and D). However, QTL distributions greatly varied from different homoeologous groups, sub-genomes and individual chromosomes (Figure 3A). For instance, the number of initial QTLs ranged from 45 (11.42%) on the homoeologous group 3 to 68 (17.26%) on the group 2, and from 6 (1.50%) on chromosome 1D to 35 (8.89%) on 2A. By comparison, more QTLs were distributed on A (165/394, 41.88%) and B sub-genomes (159/394, 40.36%), but fewer were harbored on the D sub-genome (70/394, 17.77%). In addition, these QTLs had initial CIs varying from 0.10 to 45.50 cM, with an average of 11.85 cM. There were 55.08% (217) of these 394 loci with initial CIs lower than 10 cM and 80.71% (318) with initial CIs lower than 20 cM (Figure 3B). Correspondingly, the individual PVE ranged from 1.00% to 39.70%, with an average of 9.38% (Figure 3C). Only 36.04% of loci showed the PVE values higher than 10%, indicating that most of them were minor QTLs.

### Initial Quantitative Trait Locus Projection and Identification of Meta-QTL for Thousand Grain Weight

**FIGURE 4:**
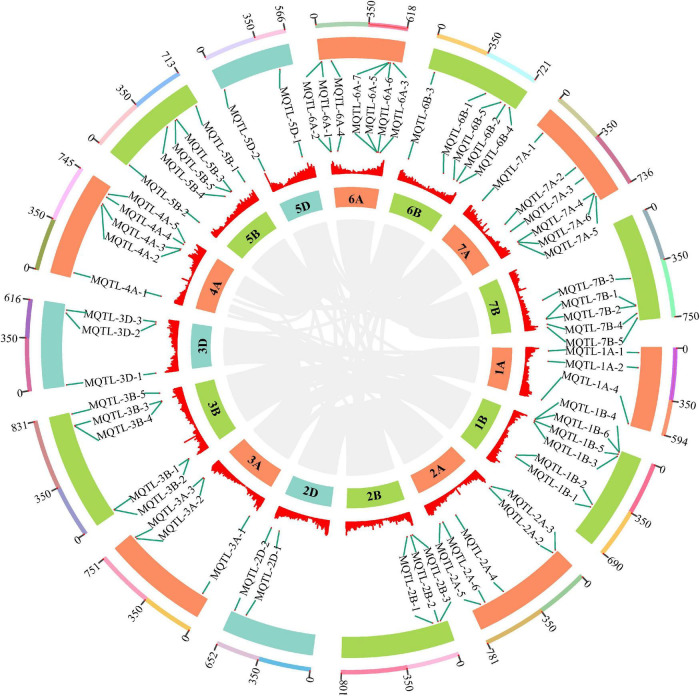
The chromosome distribution of 65 MQTLs for TGW by MQTL analysis. The circles from outside to inside represent the chromosome physical distance (Mb), 65 MQTLs position, density of high confidence genes and wheat chromosome, respectively. The connecting lines in the center of the circular diagram represents wheat genomic collinearity (gray).

Based on the above TGW QTL collection, a total of 394 initial QTL data were used to project onto the consensus map developed by integrating individual maps from 45 earlier studies into a reference genetic map. As a result, 286 QTLs were successfully mapped, while the remaining QTLs were eliminated due to the absence of their flanking markers on the consensus map. For the requirements of both the lowest model value and the minimum of ten QTLs projected on each chromosome for an accurate MQTL analysis, 274 of 286 projected QTLs were finally grouped into 67 MQTLs on chromosomes 1A, 1B, 2A, 2B, 2D, 3A, 3B, 3D, 4A, 5B, 5D, 6A, 6B, 7A and 7B (Figure 4, Supplementary Table 4, and Supplementary Figure 3). The 95% CI varied from 0.04 (MQTL-2B-3) to 30.76 cM (MQTL-3D-3), with an average CI of 3.18 cM, which was 3.73-fold less than that of initial QTLs (11.85 cM). This suggested that these MQTLs were mapped more precisely. Considering QTL distributions, each chromosome at least harbored two MQTLs and eight (5D) to 29 initially projected QTLs (2A). Based on the flanking marker sequence comparison, 65 MQTLs had definite physical positions on the wheat genome reference sequence of Chinese Spring, while the positions of two MQTLs, MQTL-1A-3 and MQTL-2A-1, were not well matched (Supplementary Table 4). The physical intervals of these 65 MQTLs ranged from 1.13 to 259.05 Mb. In particular, five core MQTLs, such as MQTL-1B-6, MQTL-2D-2, MQTL-3B-2, MQTL-6A-4, MQTL-7B-5, were positioned with the narrower physical intervals less than 20 Mb and genetic distance shorter than 1.0 cM, which fulfilled the established selection criteria for further mining putative candidate genes.

### Putative Candidate Genes Mined Within Meta-Quantitative Trait Locus Regions

**FIGURE 5:**
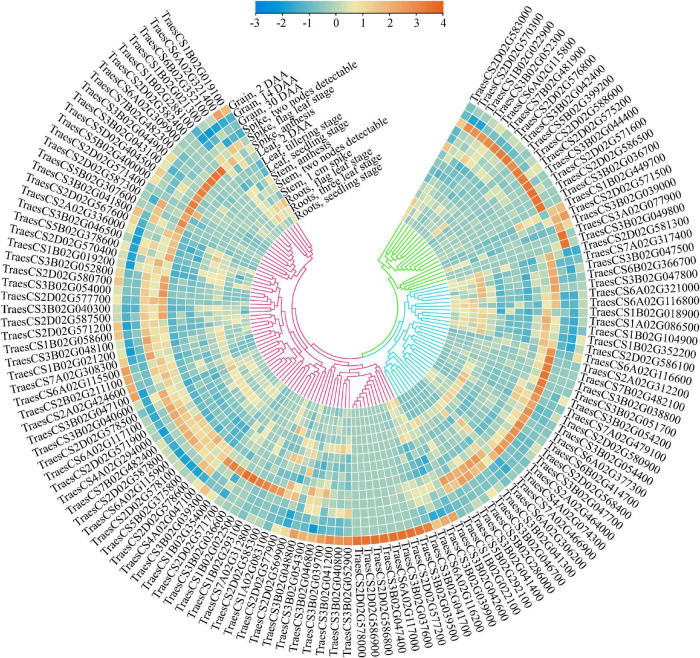
Expression characteristics of 134 putative candidate genes in five tissues. The transcriptome data was downloaded from expVIP (http://www.wheat-expression.com), and TPM value was used to characterize the expression level based on normalized scale method.

To further mine the putative candidate genes affecting wheat grain weight, a detailed search for cloned genes affecting grain weight in rice was conducted, and 180 functional genes were finally obtained. Of these, 85 genes were found in 32 MQTLs regions, with an average of 2.6 genes per MQTL (Supplementary Table 4). These genes were early reported to affect grain weight in rice through a variety of pathways, such as MYB transcription factor, zinc finger protein, gibberellin, kinase family protein, UDP-glycosyltransferase, and so on. In general, these putative candidate genes were of high confidence, and the effects of their orthologous on grain weight in rice were investigated intensively. Meanwhile, five core MQTLs, such as MQTL-1B-6, MQTL-2D-2, MQTL-3B-2, MQTL-6A-4 and MQTL-7B-5, showed a genetic distance between 0.16 and 0.66 cM; a physical distance between 1.39 and 13.9 Mb and were supported by three or seven initial QTLs. All of them fulfilled the established criteria to mine candidate genes, *i.e.*, simultaneously obtained from analysis of at least two initial QTLs, and being shorter than 1.0 cM and 20 Mb in genetic length and physical length, respectively. Using the annotation of wheat reference genome sequence of Chinese spring, there were 513 putative candidate genes mined within five core MQTLs (Supplementary Table 5). Those genes were associated with E3 ubiquitin-protein ligase, cytochrome P450 family protein, F-box family protein and zinc finger protein. To further identify more reliable genes by combining transcriptomic data, 134 putative candidate genes were found as highly and specifically expressed in the grain and/or spike (TPM > 2), with higher expression values than in other tissues (Figure 5 and Table 2). The expression patterns of these putative candidate genes could be further divided into three classes (Figure 5). Putative candidate genes in Class I was mostly expressed in the spike at the anthesis and 2-DAA grain stages. Putative candidate genes in Class II were mainly expressed in the spike at the two nodes detectable and flag leaf stages. Putative candidate genes in Class III were highly expressed in the grain at the 14 DAA and 30 DAA stages. Even in the same organ, the gene expression patterns significantly varied from different growth stages, *e.g.*, TraesCS3B02G039000, TraesCS2D02G571500, TraesCS3B02G049800, and so on. This implied that these putative candidate genes showed tissue- and development-dependent expression patterns. As a result, these crucial genes were highly and specifically expressed in grains and spikes, which could highly affect the TGW trait in wheat.

**TABLE 2:** Summary of 134 putative candidate genes exhibiting significant expression (TPM > 2) within MQTLs.

MQTL	Putative Candidate gene ID	Gene function annotation	Ortholog in rice
MQTL-1A-1	TraesCS1A02G083100	MYB transcription factor	MYB61; qNLA1; qCel1
	TraesCS1A02G086500	Mitogen-activated protein kinase	OsMPK15; OsMPK16
MQTL-1B-1	TraesCS1B02G288100	Dual specificity phosphatase	OsCOI1b
	TraesCS1B02G293100	Mitogen-activated protein kinase	OsLAC
	TraesCS1B02G352200	Coronatine insensitive 1-like protein	OsSec18
	TraesCS1B02G354000	Laccase	SLG
	TraesCS1B02G449700	ATP-dependent zinc metalloprotease FtsH	OsAGPL2; OsAPL2; shr1; GIF2
MQTL-1B-5	TraesCS1B02G058600	HXXXD-type acyl-transferase family protein	OsMKP1; GSN1
	TraesCS1B02G104900	Glucose-1-phosphate adenylyltransferase	OsMPK15; OsMPK16
MQTL-1B-6	TraesCS1B02G018900	Ras-related protein, expressed	Os05g0105100
	TraesCS1B02G019100	Ras-like protein	Os05g0105200
	TraesCS1B02G019200	Tubulin-specific chaperone cofactor E-like protein	Os05g0105300
	TraesCS1B02G021200	RNA-binding family protein	Os05g0105900
	TraesCS1B02G021300	Phosphatidate cytidylyltransferase	Os01g0758400
	TraesCS1B02G022100	NBS-LRR disease resistance protein-like protein	Os01g0547000
	TraesCS1B02G022500	Protein trichome birefringence	Os10g0254720
	TraesCS1B02G022900	Nuclear inhibitor of protein phosphatase 1	Os08g0326100
MQTL-2A-4	TraesCS2A02G312200	Zinc finger protein	NSG1; LRG1
	TraesCS2A02G336000	Aldehyde dehydrogenase	OsALDH10A5; OsBADH1
MQTL-2A-5	TraesCS2A02G464000	Alcohol dehydrogenase, putative	GSD1; gsd1-D
MQTL-2A-6	TraesCS2A02G424600	Remorin family protein	FC1; OsCAD7
MQTL-2B-3	TraesCS2B02G211100	Gibberellin regulated protein	OsGASR9
MQTL-2D-2	TraesCS2D02G568400	DNA/RNA helicase protein	ENL1
	TraesCS2D02G580700	Ubiquitin	Os06g0681400
	TraesCS2D02G580900	CsAtPR5	Os04g0689800
	TraesCS2D02G581300	RNA-binding region RNP-1	Os04g0689700
	TraesCS2D02G582400	PI-PLC X domain-containing protein	Os04g0689300
	TraesCS2D02G583000	Peroxidase	Os04g0689000
	TraesCS2D02G585300	ABC transporter G family member	Os01g0615500
	TraesCS2D02G586100	30S ribosomal protein S11	Os03g0385900
	TraesCS2D02G586500	WAT1-related protein	Os04g0687800
	TraesCS2D02G586800	Cysteine proteinase inhibitor	Os03g0429000
	TraesCS2D02G586900	Cysteine proteinase inhibitor	Os03g0429000
	TraesCS2D02G587300	Chaperone protein DnaJ	Os04g0687300
	TraesCS2D02G587500	Lectin protein kinase family protein	NA
	TraesCS2D02G587800	CsAtPR5	Os04g0689800
	TraesCS2D02G588600	Kinase family protein	Os04g0686600
	TraesCS2D02G567600	Magnesium transporter, putative (DUF803)	Os01g0882300
	TraesCS2D02G569900	S-adenosyl-L-methionine-dependent methyltransferases superfamily protein	Os04g0692400
	TraesCS2D02G570300	RING/FYVE/PHD zinc finger superfamily protein	Os04g0692300
	TraesCS2D02G570400	RING/FYVE/PHD zinc finger superfamily protein	Os04g0692300
	TraesCS2D02G571200	EamA-like transporter family protein	OsUGT1
	TraesCS2D02G571400	Chitinase	Os05g0399300
	TraesCS2D02G571500	DNA-directed RNA polymerase subunit	Os05g0151000
	TraesCS2D02G571600	Chitinase	Os03g0418000
	TraesCS2D02G571700	C2 calcium/lipid-binding and GRAM domain protein	Os04g0691800
	TraesCS2D02G571900	RING/FYVE/PHD zinc finger protein	Os04g0691700
	TraesCS2D02G575200	Chaperone DnaK	Os03g0113700
	TraesCS2D02G576800	DDB1-and CUL4-associated factor-like protein 1	Os04g0691200
	TraesCS2D02G577200	E3 ubiquitin-protein ligase RNF126-A	NA
	TraesCS2D02G577700	GEM-like protein 1	Os03g0187600
	TraesCS2D02G577900	UPF0503 protein, chloroplastic	Os04g0690500
	TraesCS2D02G578000	Carboxyl-terminal peptidase (DUF239)	Os07g0422700
	TraesCS2D02G578100	Nodulin homeobox	Os05g0188600
	TraesCS2D02G578500	cDNA clone: J033115O13, full insert sequence	Os04g0690400
	TraesCS2D02G578600	Tetratricopeptide repeat	Os04g0690300
MQTL-3A-1	TraesCS3A02G077900	NAC domain-containing protein	OsNAC20; ONAC020
MQTL-3B-2	TraesCS3B02G038800	NADP dependent sorbitol 6-phosphate dehydrogenase family protein	Os02g0123500
	TraesCS3B02G039000	Mannose-6-phosphate isomerase	Os01g0127900
	TraesCS3B02G039300	Protein DETOXIFICATION	Os10g0195000
	TraesCS3B02G039500	Nuclease S1	Os01g0128100
	TraesCS3B02G039700	Nuclease S1	Os01g0128200
	TraesCS3B02G039900	Transmembrane protein 214	Os01g0128400
	TraesCS3B02G040300	DUF1666 family protein	Os01g0129500
	TraesCS3B02G040600	DNA-binding storekeeper protein-related transcriptional regulator	Os02g0288200
	TraesCS3B02G040800	Protein NEGATIVE REGULATOR OF RESISTANCE	NRR
	TraesCS3B02G041200	Protein NEGATIVE REGULATOR OF RESISTANCE	NRR
	TraesCS3B02G041300	Disease resistance protein RPM1	Os11g0265900
	TraesCS3B02G041400	Disease resistance protein (NBS-LRR class) family	Os02g0272900
	TraesCS3B02G041700	Alpha-glucosidase	Os01g0130400
	TraesCS3B02G041800	Translation initiation factor IF-2	Os01g0130900
	TraesCS3B02G036600	Dihydroflavonol-4-reductase	Os01g0127500
	TraesCS3B02G036700	Bowman-Birk type trypsin inhibitor	Os01g0127600
	TraesCS3B02G037600	Bowman-Birk type trypsin inhibitor	Os01g0127600
	TraesCS3B02G042400	AP2-EREBP transcription factor	Os01g0131600
	TraesCS3B02G042600	Signal peptidase subunit family protein	Os01g0131800
	TraesCS3B02G043100	Mitochondrial import inner membrane translocase subunit TIM22	Os04g0405100
	TraesCS3B02G044400	Beta-galactosidase 8	NA
	TraesCS3B02G044900	Cytochrome P450 family protein, expressed	Os03g0138200
	TraesCS3B02G046500	Deoxyhypusine synthase	Os03g0740600
	TraesCS3B02G046700	Receptor-like kinase	Os01g0133900
	TraesCS3B02G046800	Eukaryotic translation initiation factor 4E	Os01g0970400
	TraesCS3B02G051700	B3 domain-containing protein Os01g0723500	NA
	TraesCS3B02G052300	E3 ubiquitin-protein ligase	Os01g0125000
	TraesCS3B02G052800	E3 ubiquitin-protein ligase	Os05g0152900
	TraesCS3B02G052900	Glycosyltransferase	OsGT61-1; XAX1
	TraesCS3B02G054000	E3 ubiquitin-protein ligase	Os01g0122200
	TraesCS3B02G054200	E3 ubiquitin-protein ligase	Os01g0121900
	TraesCS3B02G054300	E3 ubiquitin-protein ligase	Os01g0122200
	TraesCS3B02G054400	E3 ubiquitin-protein ligase	Os01g0121900
	TraesCS3B02G047100	Hydroxyacylglutathione hydrolase	Os01g0133500
	TraesCS3B02G047400	Carboxyl-terminal peptidase (DUF239)	Os07g0573400
	TraesCS3B02G047500	RuvB-like helicase	Os07g0178900
	TraesCS3B02G047700	Cotton fiber-like protein (DUF761)	Os01g0133200
	TraesCS3B02G047800	Hexose transporter	Os01g0133100
	TraesCS3B02G048100	Mediator of RNA polymerase II transcription subunit 22	Os01g0132700
	TraesCS3B02G048800	Heat-shock protein	Os01g0135900
	TraesCS3B02G049800	Heat shock protein	Os01g0136100
MQTL-4A-1	TraesCS4A02G047100	Activating signal cointegrator 1 complex subunit 2	SPL35
	TraesCS4A02G074300	GAGA-binding transcriptional activator	OsGBP3
MQTL-4A-2	TraesCS4A02G294000	Guanine nucleotide-binding protein subunit beta	OsRGB1
MQTL-5B-3	TraesCS5B02G375800	Squamosa promoter binding-like protein	GL3.1; qGL3-1; qGL3; OsPPKL1
	TraesCS5B02G378600	Basic helix-loop-helix (bHLH) DNA-binding superfamily protein	LO9-177
	TraesCS5B02G399200	Serine carboxypeptidase, putative	GSA1; UGT83A1
	TraesCS5B02G400000	Serine/threonine-protein phosphatase	OsPho1
MQTL-5B-4	TraesCS5B02G286000	KxDL motif protein	OsSPL18
	TraesCS5B02G292100	UDP-glycosyltransferase	OsBC1
	TraesCS5B02G307600	Alpha-1,4 glucan phosphorylase	OsSCP46
MQTL-5D-1	TraesCS5D02G404500	Alpha-1,4 glucan phosphorylase	OsPho1
MQTL-6A-4	TraesCS6A02G115500	S-adenosyl-L-methionine-dependent methyltransferases superfamily protein	NA
	TraesCS6A02G115800	DNA/RNA-binding protein KIN17	Os03g0570300
	TraesCS6A02G115900	Splicing factor 3B subunit 1	OsSF3B1
	TraesCS6A02G116200	ATP-dependent RNA helicase	OseIF4A
	TraesCS6A02G116600	Polynucleotide 5’-hydroxyl-kinase NOL9	Os01g0354700
	TraesCS6A02G116800	Harpin-induced protein	Os07g0250501
	TraesCS6A02G117000	HLA class II histocompatibility antigen, DRB1-16 beta chain	NA
	TraesCS6A02G117100	DNA-binding protein BIN4	Os02g0147700
MQTL-6A-5	TraesCS6A02G306200	Transcription factor protein	PGL2; OsBUL1
	TraesCS6A02G309900	Peroxidase	YPD1
	TraesCS6A02G321000	Plant cadmium resistance 2	qTGW2; OsCNR1
	TraesCS6A02G321400	Cyclin-dependent kinase inhibitor	OsKRP1
MQTL-6A-7	TraesCS6A02G377300	LIGHT-DEPENDENT SHORT HYPOCOTYLS-like protein (DUF640)	TH1; BSG1; BLS1; AFD1; BH1
MQTL-6B-5	TraesCS6B02G352100	Cyclin-dependent kinase inhibitor	OsKRP1
	TraesCS6B02G366700	Growth-regulating factor	OsGRF1; rhd1
	TraesCS6B02G414700	LIGHT-DEPENDENT SHORT HYPOCOTYLS-like protein (DUF640)	TH1; BSG1; BLS1; AFD1; BH1
MQTL-7A-2	TraesCS7A02G308300	Fertilization independent endosperm 1 protein	OsFIE1; Epi-df
	TraesCS7A02G312800	Amino acid transporter, putative	OsHT; OsLHT1
	TraesCS7A02G317400	IQ domain-containing protein	OsIQD14
	TraesCS7A02G466900	DNA primase/helicase	TWINKLE
	TraesCS7A02G479100	PLATZ transcription factor family protein	GL6; SG6
MQTL-7B-5	TraesCS7B02G481900	F-box family protein	Os07g0120200
	TraesCS7B02G482100	Protein argonaute	OsAGO1c
	TraesCS7B02G482300	NBS-LRR disease resistance protein	Os02g0456800
	TraesCS7B02G482400	Chromodomain-helicase-DNA-binding family protein	CHR723

**FIGURE 6:**
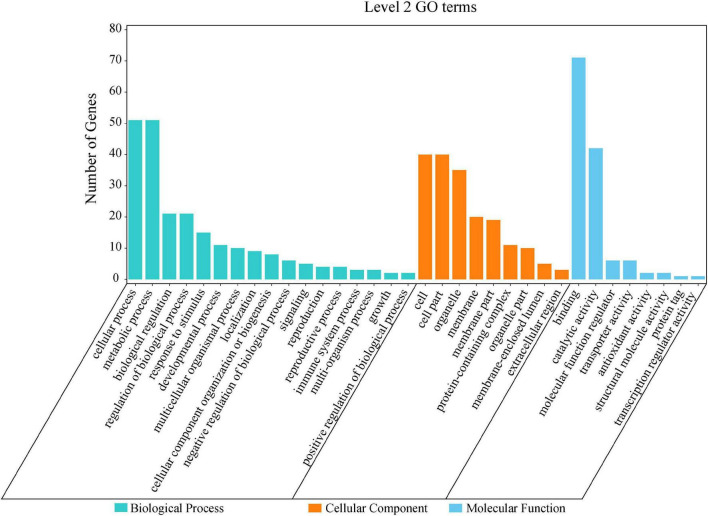
Level 2 GO terms for 134 putative candidate genes from MQTL regions.

**FIGURE 7:**
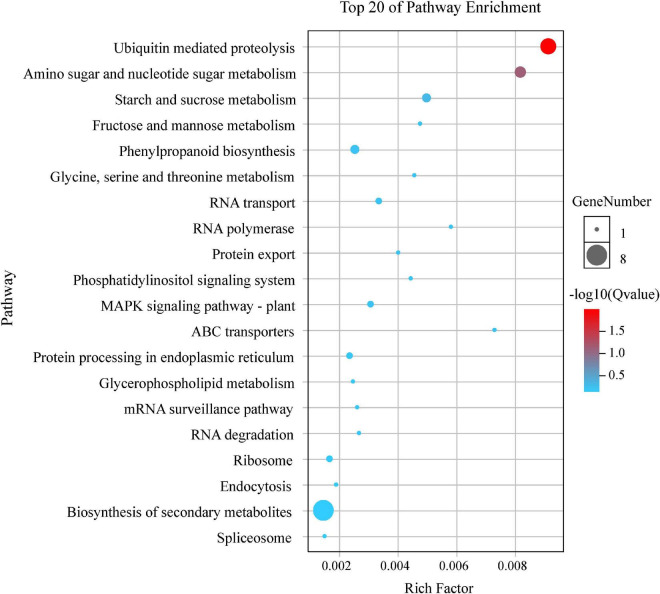
Top 20 KEGG enrichment pathways for 134 putative candidate genes from MQTL regions.

By the GO and KEGG analysis, these genes were mainly associated with biological process (17 sub-functions), cellular component (9 sub-functions) and molecular function (8 sub-functions) (Figure 6). The most significantly enriched GO terms associated with biological process were for cellular (51/134, 38.06%) and metabolic (51/134, 38.06%). In terms of cellular component, the genes were enriched mainly in cell (40/134, 29.85%) and cell part (40/134, 29.85%). In KEGG pathways, these putative candidate genes were highly involved in the pathways of ubiquitin mediated proteolysis, amino sugar and nucleotide sugar metabolism and starch and sucrose metabolism (Figure 7).

## Discussion

### Quantitative Trait Locus Identification and Stable Quantitative Trait Locus Comparisons for Thousand Grain Weight

Thousand Grain Weight is a key determinant that is related to grain yield potential in wheat and is influenced by both genetic and environmental factors (Wang L. et al., 2012; Avni et al., 2018; Duan et al., 2020). Compared with other yield components, TGW had more stable phenotypic variation and higher heritability (Mir et al., 2012; Wang L. et al., 2012; Cheng et al., 2015; Hu et al., 2015; Krishnappa et al., 2017; Avni et al., 2018; Kumari et al., 2018; Duan et al., 2020; Xin et al., 2020; Yang et al., 2020; Gao et al., 2021; Qu et al., 2021). Likewise, TGW trait in the present study also showed a prominent main-effect of genotype, with a high heritability (*h*^2^_B_ = 0.77), relatively lower CV% (6.08-12.80%), and significant correlations (*r* = 0.62^**^−0.92^**^) in the performance of the RILs under eight different environments. This confirmed that TGW was predominantly controlled by the genetic factor, and suggested the role of QTLs expressed across environments (Figure 1 and Table 1). Indeed, some stable QTLs with relatively higher PVE (6.65-12.87%) were identified in the present RIL population (Figure 2 and Supplementary Table 2). However, in this study, 25 of 45 QTLs were identified in specific environments and most of them explained lower PVE (1.76-8.79%), implying that these QTLs were expressed sensitively to individual environments. Similar results have also been found in other early studies, where GEI effects and epistatic effects significantly influenced TGW genetic variation to some extent (Hu et al., 2015; Kumari et al., 2018; Qu et al., 2021). It was also interpreted why some loci identified with minor-effects were always erratic and highly responsible for individual environments. This suggested that in addition to additive effect, GEI and epistatic effects should not be ignored in TGW genetic improvement.

In this study, ten stable QTLs were significantly expressed in at least four environments (Supplementary Table 2). These crucial QTLs were distributed on chromosomes 1A, 1B, 2A, 2B, 4A, 5B, 6B, 7B and 7D, accounting for the PVE from 6.65% to 12.87% higher than other identified loci explained. In particular, several stable QTLs detected in this study shared similar chromosomal positions or regions with other detected earlier. For example, a present stable QTL, *Qtgw.acs-1A.3*, highly adjacent to the marker Xgwm99 on 1A, was identified and verified earlier as a major and stable QTL for grain weight (*QGw.ccsu-1A.3*) by combining linkage mapping and association mapping methods. The marker Xgwm99 was also suggested as a functional marker to be used in MAS for TGW (Mir et al., 2012). The marker intervals of three stable QTLs, *Qtgw.acs-4A.2*, *Qtgw.acs-5B.3*, *Qtgw.acs-6B.1*, were overlapped to those of some minor-effect QTLs for TGW identified earlier (Groos et al., 2003; Wang et al., 2009; Wang et al., 2010; He et al., 2020), while *Qtgw.acs-5B.1* was highly adjacent to the marker interval with a stable QTL cluster for TGW and grain width reported by Ramya et al. (2010). The location of a stable QTL, *Qtgw.acs-1B.1*, in the marker interval Xgwm413-Xwmc419 on 1B, was similar to the location of several clustered QTLs for grain yield-related traits reported by Peng et al. (2003). Similarly, *Qtgw.acs-2A.1* and *Qtgw.acs-2B* were mapped to a similar position to other reported loci for heading time (Fayt et al., 2011), plant height and spike traits (Wang et al., 2014; Hu et al., 2019), owing to proximity to Xgwm512 on 2A and Xgwm429 on 2B, respectively. A stable QTL, *Qtgw.acs-7D.1*, located in the marker interval of Xgwm635-Xgwm428 on 7D, was highly overlapped to the positions of several major and stable loci for grain size (Yan et al., 2017) and sterile spikelet number identified earlier (Ma et al., 2007). This indicated that above-mentioned QTLs for TGW seemed highly collocated or adjacent to those for some grain yield-related traits. Indeed, it still remains a puzzling question whether these clustered QTLs represent close linkages of multiple genes affecting different traits or have pleiotropic effects of regulatory genes that affect the related traits. Besides, a stable QTL, *Qtgw.acs-7B.1*, was detected only in this study and could be a novel locus. These stable and common QTL, as well as closely linked molecular markers, were therefore suggested with a great potential in MAS to improve TGW, along with yield potential in wheat.

### Genetic Architecture of Thousand Grain Weight Revealed by Meta-QTL Analysis

To further dissect the genetic architecture of TGW trait in the present study, MQTL analysis was performed using reported QTLs from previous mapping studies and identified QTLs in the present study. As a result, 394 initial QTLs were successfully collected and further employed for MQTL analysis (Figure 3 and Supplementary Table 3). These loci were unevenly distributed on 21 chromosomes, varying from six QTLs on 1D to 35 QTLs on 2A. By comparison, about 82.2% of initial QTLs were harbored on A and B sub-genomes. The result was consistent with previous MQTL analysis for grain yield and yield-related traits, where 72.1%-86.2% of initial loci were reported in A and B sub-genomes (Acuña-Galindo et al., 2015; Tyagi et al., 2015; Kumar et al., 2020; Liu H. et al., 2020; Yang et al., 2021). This implied that these QTLs were located more on the A and B sub-genomes, but fewer were on D sub-genome. It could be attributed to the low level of polymorphism in the D sub-genome of hexaploid wheat. Since the D-genome is a recent evolutionary addition to the hexaploid wheat genome, there has been limited gene flow from *Aegilops tauschii* to cultivated wheat, resulting in a relatively narrow genetic variation (Kumar et al., 2012). Although fewer TGW QTLs and MQTLs were identified on chromosomes 2D, 3D, and 5D in this study, it was still noteworthy that some useful QTL/genes mainly controlling desirable traits have been discovered on the D sub-genome, including abiotic and biotic stress tolerance and TGW-related traits in wheat (Kumar et al., 2012; Yan et al., 2017).

Meta-QTL analysis can refine QTL locations in different genetic backgrounds and environments, providing more accurate genomic regions associated with target traits (Goffinet and Gerber, 2000; Semagn et al., 2013; Acuña-Galindo et al., 2015; Soriano and Alvaro, 2019; Kumar et al., 2020; Liu H. et al., 2020; Liu Y. et al., 2020; Yang et al., 2021). In this study, 274 initial QTLs were finally grouped into 67 MQTLs on chromosomes 1A, 1B, 2A, 2B, 2D, 3A, 3B, 3D, 4A, 5B, 5D, 6A, 6B, 7A and 7B (Figure 4 and Supplementary Table 4). The average 95% CI of MQTLs (3.18 cM) was 3.73-fold less than that of initial QTLs (11.85 cM). A similar result was reported earlier by Yang et al. (2021), where the average CI of identified MQTLs for yield-related traits was 2.9 times less than that of initial QTLs. By the peak marker sequences compared with the wheat genome reference sequence of Chinese Spring, 65 MQTLs had definite physical positions and the physical intervals ranged from 1.13 to 259.05 Mb. However, some MQTLs were excluded from further elucidation, because they only harbor singular QTL. For these under-represented QTLs, more loci should be added to analyze the responsibility of these regions for TGW (Venske et al., 2019).

Among 67 MQTLs identified, five core MQTLs, such as MQTL-1B-6, MQTL-2D-2, MQTL-3B-2, MQTL-6A-4 and MQTL-7B-5, fulfilled the criteria with narrower physical intervals (< 20 Mb) (Supplementary Table 4), shorter genetic distance (< 1.0 cM) and more initial QTLs (*n* ≥ 2) (Venske et al., 2019). Therefore, these MQTLs will be highly favorable for future MAS in TGW improvement, and for isolating key genes by the map-based cloning approach in wheat (Kumar et al., 2020; Liu H. et al., 2020). In addition, since five core MQTLs comprised initial QTLs detected in quite diverse and various segregating populations, the probability of involvement of the genomic regions in the regulation of target phenotype in new genetic backgrounds increases (Ribaut and Ragot, 2007; Löffler et al., 2009). For example, MQTL-2D-2 was formed by seven initial QTLs with average PVE of 8.29% from five different populations (Huang et al., 2003; Cuthbert et al., 2008; Wang et al., 2009; Wu et al., 2011; Zhang et al., 2019). MQTL-3B-2 covered five initial QTLs with average PVE of 5.68% from four different populations (Huang et al., 2006; Cuthbert et al., 2008; Wu et al., 2015) (including the population in this study). MQTL-7B-5 contained two initial QTLs with average PVE of 8.20% from two different populations (Shukla et al., 2015; Guan et al., 2018). In particular, the accuracy and validity of MQTL-1B-6 and MQTL-6A-4 would be further increased when the number of observed QTL was at least five (Zhang et al., 2017) and had high PVE (Löffler et al., 2009). MQTL-1B-6 was comprised of five initial QTLs with average PVE of 13.11% from five different populations (Yu et al., 2014; Roncallo et al., 2017; Guan et al., 2018; Goel et al., 2019) (including the population in this study). MQTL-6A-4 was composed of five initial QTLs with average PVE of 12.44% from four different populations (Peleg et al., 2011; Mir et al., 2012; Guan et al., 2018; Goel et al., 2019). They contained more QTL from different populations with high PVE, indicating that these crucial MQTLs had more extensive adaptability in TGW improvement (Kumar et al., 2020; Yang et al., 2021). In addition, MQTL-1B-6 and MQTL-3B-2 overlapped the physical positions of MQTLs for wheat yield-related traits detected in recent studies (Liu H. et al., 2020; Yang et al., 2021). This further confirmed the reliability of present MQTLs, which would be highly favorable for future MAS in TGW improvement, and for isolating key genes by the map-based cloning approach in wheat (Kumar et al., 2020; Liu H. et al., 2020).

### Putative Candidate Genes for Thousand Grain Weight Mined in Meta-QTL Regions

In order to obtain reliable candidate genes, two strategies were combined to screen for candidate genes. On the one hand, it was feasible to screen important candidate genes by the interspecific homology analysis. In this context, candidate genes might be confirmed to have rice homologs with similar function in wheat, because these development pathways were conserved among related grass species (Li and Li, 2016; Liu et al., 2017). On the other hand, given the fundamental differences in seed development, not all gene functions were conserved (Brinton and Uauy, 2019). Thus, the method proposed by Venske et al. (2019) was used as a supplement to fully mine candidate genes. Among 67 MQTLs identified in this study, five core MQTLs, such as MQTL-1B-6, MQTL-2D-2, MQTL-3B-2, MQTL-6A-4, and MQTL-7B-5, meet the above-mentioned criteria (Supplementary Table 4).

Whilst most stages of grain development had been widely characterized phenotypically, the genetic basis how these processes were controlled and how they affected final grain weight was not well known in wheat. (Brinton and Uauy, 2019). Understanding gene expression patterns were favorable to narrow down candidate genes within a defined genetic interval (Borrill et al., 2016). Meanwhile, transcriptomic and omics studies provided a global overview of the types of genes involved in grain development (Brinton and Uauy, 2019). Therefore, the data from expVIP platform would facilitate the meta-analysis and easily allow integration of data for candidate gene expression analysis (Borrill et al., 2016). Analysis of putative candidate genes in the MQTL intervals were conducted, including diverse developmental time courses and tissues underlying expVIP platform, GO and KEGG enrichment. Those can deepen our understanding of differential expression and regulative mechanisms, in order to prioritize candidate genes (Zheng et al., 2020). Herein, putative candidate genes presenting over 2 TPM were only considered (Wagner et al., 2013; Venske et al., 2019). A total of 134 genes were found with high and/or specifical expression patterns in the grain and/or spike (TPM > 2) (Figure 5). Herein, these putative candidate genes showed tissue- and development-dependent expression patterns, which could highly affect the TGW trait in wheat. For example, TraesCS3A02G077900, encoding NAC domain-containing protein, was specifically expressed in grain 14 DAA. In rice, its homologous genes *OsNAC20*, as a NAC transcription factor, significantly decreased starch and storage protein content by OsNAC20/26 double mutant, and the phenotype was characterized by a significant reduction in TGW (Wang et al., 2020). TraesCS3B02G051700, encoding a B3 domain-containing protein from a large B3 transcription factor superfamily, was highly expressed in spike two nodes detectable stage. It has been demonstrated that B3 superfamily plays a central role in the embryogenesis to seed maturation and dormancy of the plant (Wang Y. et al., 2012). TraesCS2D02G571200, encoding EamA-like transporter family protein, was widely expressed in wheat tissue. Li et al. (2018) found that EamA-like transporter as an auxin transporter required for auxin homeostasis was significantly associated with yield and yield-related traits by GWAS. Those genes deserved further study to unveil their possible role in TGW and their application in breeding programs. Although the relationship between these genes with grain development in wheat has not been reported, several homologous genes have been shown to participate in the regulation of TGW in rice, such as *OsSec18*, *OsBADH1*, and *OsLHT1*, indicating that these 134 putative candidate genes could be involved in TGW regulation in wheat (Tang et al., 2014; Sun et al., 2015; Guo et al., 2020).

GO enrichment and KEGG analysis for differentially-expressed genes in MQTL intervals provided new insights into the genetic control of TGW (Zheng et al., 2020). Herein, these putative candidate genes were highly involved in the pathways of ubiquitin mediated proteolysis, amino sugar and nucleotide sugar metabolism and starch and sucrose metabolism (Figure 7). Likewise, early studies had found that the ubiquitin proteolytic system played an important role in a broad array of basic cellular processes. For instance, Zhang et al. (2018) reported *TaGW2* encoded an E3 ubiquitin ligase and had two homeologs, *TaGW2-B1* and *TaGW2-D*, both of which were highly associated with the genetic control of grain weight in wheat. Starch was the main component of wheat grains, so it was considered as a key determinant of wheat yield, and sucrose and starch metabolism might be correlated with increasing grain yield (Guan et al., 2019).

In summary, this implied that these above-mentioned genes could directly or indirectly participate in the regulation of wheat grain development, and ultimately affect grain weight formation. Once these putative candidate genes were successfully cloned and verified in the future, they would increase our understanding of the complex molecular mechanisms underlying TGW and provide a great application potential in the molecular breeding for TGW in wheat.

## Conclusion

In this study, a total of 45 TGW QTLs were identified using a RIL population, where ten loci were highly stable across more than four environments. By the MQTL analysis, 274 of 394 initial QTLs were successfully refined into 67 MQTLs for TGW. The average confidence interval of these MQTLs was 3.73-fold less than that of initial QTLs. This suggested that the present MQTLs were mapped more precisely. In particular, five core MQTL regions were positioned in narrower genetic distance (< 1 cM) and physical distance (< 20 Mb). Putative candidate genes were mined by genomic sequence comparison to that of Chinese Spring wheat reference genome. Crucial genes were involved in three pathways of ubiquitin mediated proteolysis, amino sugar and nucleotide sugar metabolism, and starch and sucrose metabolism. Some of the genes had similar functions to those reported earlier for grain development and gran weight formation. This suggested that the key genes would have a great application potential in the molecular breeding for TGW in wheat.

## Data Availability Statement

Publicly available datasets were analyzed in this study. The data presented in the study are deposited in the figshare repository, accession number https://doi.org/10.6084/m9.figshare.17163548.v2.

## Author Contributions

YM and DY conceived of the study. YM, FJ, JM, and ZC performed phenotypic data measurement and data analysis. FJ, YL, PZ, and TC prepared figures and provided scientific feedback and revised the content. YM wrote the first draft of the manuscript. DY reviewed and edited the manuscript. All authors have read and agreed to the published version of the manuscript.

## Conflict of Interest

The authors declare that the research was conducted in the absence of any commercial or financial relationships that could be construed as a potential conflict of interest.

## Publisher’s Note

All claims expressed in this article are solely those of the authors and do not necessarily represent those of their affiliated organizations, or those of the publisher, the editors and the reviewers. Any product that may be evaluated in this article, or claim that may be made by its manufacturer, is not guaranteed or endorsed by the publisher.

## Footnotes

1
http://www.fao.org/faostat/

2
http://wheatomics.sdau.edu.cn

3
https://wheat.pw.usda.gov/GG3

4
https://www.diversityarrays.com

5
https://www.ricedata.cn/

6
http://wheat.cau.edu.cn/TGT/

7
http://www.wheat-expression.com

8
https://github.com/CJ-Chen/TBtools/releases

9
https://www.omicshare.com/tools/

## Supplementary Materials

Supplementary Figure 1 The rainfall for each growing season in the eight tested environments. E1-E6, experimental environments at Yuzhong farm station in six years from 2013 to 2018, respectively; E7 and E8, experimental environments at Tongwei farm station in 2017 and 2018, respectively.

Supplementary Figure 2 Regression analysis for TGW based on annual rainfall. **P* < 0.05.

Supplementary Figure 3 Distribution and location of 67 MQTLs on 15 different chromosomes in wheat. The horizontal line on the left of the chromosomes means the successive changes in the phenotypic variance explained (PVE) of individual QTLs and the vertical lines shows the confidence intervals of individual QTLs. And right side on the chromosomes means the markers genetic distance and markers, respectively.
