# Supplementary figures and images for: Major Genomic Regions for Wheat Grain Weight as Revealed by QTL Linkage Mapping and Meta-Analysis
Source: Front Plant Sci. 2022 Feb 10;13:802310. doi: 10.3389/fpls.2022.802310 (PMC8866663; doi:10.3389/fpls.2022.802310)

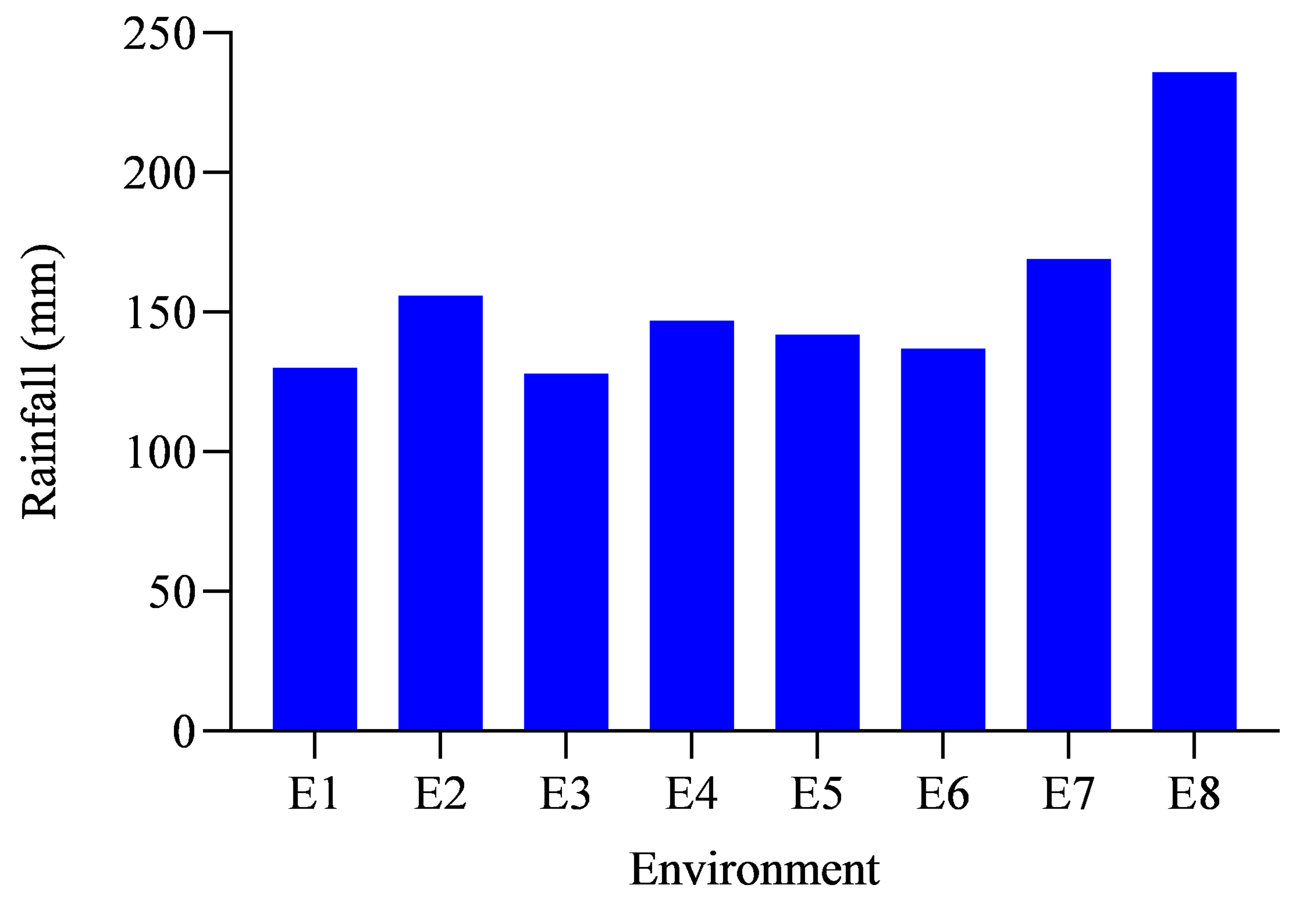

Supplement: Supplementary Figure 1 — The rainfall for each growing season in the eight tested environments. E1-E6, experimental environments at Yuzhong farm station in six years from 2013 to 2018, respectively; E7 and E8, experimental environments at Tongwei farm station in 2017 and 2018, respectively. [file Image_1.TIF]

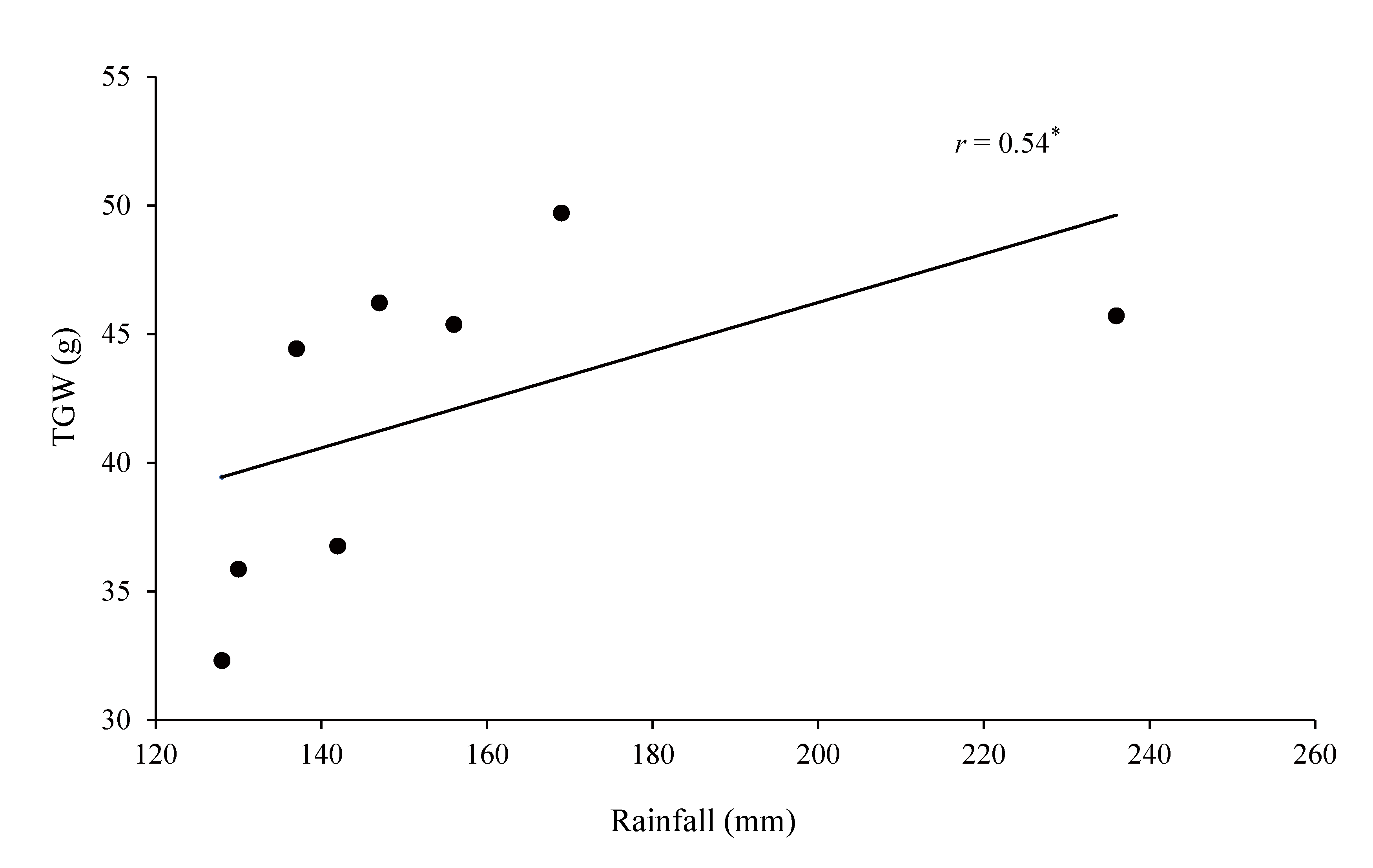

Supplement: Supplementary Figure 2 — Regression analysis for TGW based on annual rainfall. *P < 0.05. [file Image_2.TIF]
