# Supplementary material for: Major Genomic Regions for Wheat Grain Weight as Revealed by QTL Linkage Mapping and Meta-Analysis
Source: Front Plant Sci. 2022 Feb 10;13:802310. doi: 10.3389/fpls.2022.802310 (PMC8866663; doi:10.3389/fpls.2022.802310)
Supplement: Supplementary file 4 [file Data_Sheet_1.DOCX]

**SUPPLEMENTAL MATERIALS**

**SUPPLEMENTARY TABLE 1 |** Mean squares of analysis of variance (ANOVA) for TGW in the wheat RIL population

| **Source of variation** | **df** | **MS** | ***F*** |
| --- | --- | --- | --- |
| Environment (E) | 7 | 12246.14 | 12613.63** |
| Genotype (G) | 119 | 91.15 | 93.87** |
| E×G | 833 | 27.62 | 28.44** |
| Error | 1712 | 0.97 |  |
| *h^2^*_B_ | 0.77 |  |  |

RIL, recombinant inbred line; TGW, thousand grain weight; df, degrees of freedom; MS, mean square; *F*, *F* test; *h^2^*_B_, broad-sense heritability; **P* < 0.05, ** *P* < 0.01.

**SUPPLEMENTARY TABLE 2 |** Additive effects of QTLs identified for TGW in the wheat RIL population under eight tested environments and BLUP analysis

| **QTL** | **Environments** | **Marker interval** | **Site (cM)** | **LOD** | **PVE (%)** | **Add** |
| --- | --- | --- | --- | --- | --- | --- |
| *Qtgw.acs-1A.1* | E2 | Xwmc20–Xbarc240 | 61 | 4.05 | 6.02 | 1.27 |
| *Qtgw.acs-1A.2* | E8 | Xpsp3027–Xwmc337 | 88 | 3.16 | 4.08 | 0.85 |
| *Qtgw.acs-1A.3* | E1, E2, E7, E8, BLUP | Xcfa2219–Xgwm99 | 131 | 2.92, 3.63, 3.27, 3.39, 3.75 | 7.77, 9.26, 7.78, 9.82, 9.98 | -0.55, -0.72, -0.80, -0.89, -0.88 |
| *Qtgw.acs-1B.1* | E1, E2, E5, E6, E8, BLUP | Xgwm413–Xwmc419 | 7 | 3.50, 4.08, 3.53, 2.53, 2.74, 4.01 | 6.78, 6.74, 9.33, 11.21, 7.77, 10.28 | -0.56, -0.66, -0.53, -0.73, -0.24, -0.68 |
| *Qtgw.acs-1B.2* | E5 | Xgwm11–Xwmc626 | 38 | 3.03 | 3.29 | 0.73 |
| *Qtgw.acs-1B.3* | E5, E7, BLUP | Xbarc181–Xbarc61 | 62 | 3.38, 4.01, 3.66 | 9.84, 9.88, 11.75 | -0.79, -0.65, -0.53 |
| *Qtgw.acs-2A.1* | E1, E2, E3, E4, E6, BLUP | Xgwm512–Xgwm30 | 5 | 3.74, 3.24, 3.55. 3.33, 2.51, 2.63 | 12.87, 9.91, 9.82, 8.74, 9.32, 8.62 | -1.29, -0.79, -0.71, -0.88, -0.93, -0.64 |
| *Qtgw.acs-2A.2* | E2 | Xgwm122–Xmag2150 | 50 | 3.86 | 5.68 | 1.23 |
| *Qtgw.acs-2B* | E2, E3, E6, E7 | Xgwm429–Xbarc1072 | 8 | 4.23, 3.66, 5.27, 4.98 | 10.32, 7.64, 11.06, 8.32 | -1.01, -0.66, -0.95, -0.68 |
| *Qtgw.acs-3A.1* | E1 | Xmag4305–Xwmc532 | 18 | 3.30 | 4.27 | -1.34 |
| *Qtgw.acs-3A.2* | E2, E4, BLUP | Xwmc50–Xksum222 | 56 | 4.69, 3.50, 4.38 | 10.44, 9.54, 8.20 | 0.98, 0.73, 0.67 |
| *Qtgw.acs-3A.3* | E1 | Xbarc1113–Xwmc153 | 116 | 3.10 | 3.64 | -1.23 |
| *Qtgw.acs-3B.1* | E3 | Xwmc231–Xbarc173 | 43 | 3.55 | 5.22 | -1.15 |
| *Qtgw.acs-3B.2* | E5, BLUP | Xpsp3112–Xgwm72 | 53 | 3.56, 3.35 | 4.58, 4.14 | -0.86, -0.55 |
| *Qtgw.acs-3B.3* | E8 | Xwmc51–Xpsp3144 | 76 | 3.18 | 4.17 | 0.87 |
| *Qtgw.acs-3B.4* | E3 | Xksum45–Xwmc510 | 125 | 2.50 | 1.76 | -0.66 |
| *Qtgw.acs-4A.1* | E1 | Xwmc757–Xgwm610 | 34 | 4.45 | 8.79 | 0.92 |
| *Qtgw.acs-4A.2* | E2, E3, E5, E6, E7, BLUP | Xgwm610–Xgwm397 | 43 | 3.14, 6.23, 5.54, 4.52, 6.05, 5.73 | 9.54, 11.65, 9.24, 9.26, 10.11, 12.11 | 0.51, 0.41, 0.45, 0.55, 0.66, 0.79 |
| *tgw.acs-4A.3* | E2, E3, E5 | Xgwm160–Xwmc497 | 106 | 3.97, 5.77, 6.54 | 6.73, 9.22, 10.54 | -1.11, -0.72, -1.34 |
| *Qtgw.acs-4B.1* | E3 | Xbarc90–Xgwm540 | 14 | 3.03 | 3.60 | -0.96 |
| *Qtgw.acs-4B.2* | E3 | Xgwm495–Xgwm251 | 77 | 3.27 | 4.46 | 1.05 |
| *Qtgw.acs-4B.3* | E2 | Xbarc60–Xgwm6 | 105 | 3.19 | 4.05 | -1.05 |
| *Qtgw.acs-4D.1* | E1 | Xwmc617–Xpsp3007 | 4 | 3.66 | 10.36 | 1.50 |
| *Qtgw.acs-4D.2* | E6 | Xbarc48–Xgwm624 | 90 | 3.09 | 10.96 | 0.53 |
| *Qtgw.acs-5A.1* | E2 | Xgwm639–Xwmc492 | 55 | 3.52 | 9.76 | -1.10 |
| *Qtgw.acs-5A.2* | E3, E4 | Xwmc630–Xmag4263 | 95 | 3.15, 4.02 | 10.23, 12.25 | 0.72, 1.08 |
| *Qtgw.acs-5A.3* | E3, E5, BLUP | Xgwm443–Xcfa2155 | 100 | 3.69, 4.82, 5.32 | 9.65, 10.73, 12.01 | 1.21, 0.96, 1.95 |
| *Qtgw.acs-5B.1* | E1, E2, E4, E6 | Xgwm499–Xwmc734 | 39 | 2.89, 3.80, 4.77, 4.22 | 8.86, 12.23, 11.70, 10.84 | 0.61, 1,07, 1.22, 0.97 |
| *Qtgw.acs-5B.2* | BLUP | Xwmc734–Xwmc235 | 50 | 3.17 | 4.38 | 0.56 |
| *Qtgw.acs-5B.3* | E1, E3, E4, E6 | Xbarc59–Xbarc232 | 65 | 3.31, 2.98, 3.13, 2.54 | 7.88, 6.79, 6.65, 7.81 | 0.66, 0.75, 0.37, 0.44 |
| *Qtgw.acs-5D.1* | E7 | Xbarc205–Xgwm232 | 6 | 3.49 | 4.40 | -0.75 |
| *Qtgw.acs-5D.2* | E1, E3, E4, BLUP | Xbarc110–Xwmc161 | 60 | 3.94, 3.77, 4.23, 3.40 | 9.69, 7.76, 9.88, 4.61 | -0.80, -0.67, -0.64, -0.54 |
| *Qtgw.acs-6A.1* | E4, E5, E7, BLUP | Xbarc1165–Xksum157 | 33 | 4.21, 4.01, 3.89, 3.21 | 11.78, 9.07, 9.93, 7.54 | -0.99, -0.71, -0.50, -0.65 |
| *Qtgw.acs-6A.2* | E4 | Xksum255–Xbarc171 | 82 | 3.16 | 3.48 | 0.62 |
| *Qtgw.acs-6A.3* | E7, E8 | Xbarc171–Xgwm427 | 86 | 3.13, 3.16 | 3.25, 4.16 | 0.65, 0.86 |
| *Qtgw.acs-6A.4* | E3 | Xwmc621–Xbarc107 | 102 | 3.82 | 5.98 | -1.22 |
| *Qtgw.acs-6B.1* | E2, E3, E5, E6, E8, BLUP | Xgwm193–Xgwm361 | 45 | 4.37, 4.51, 2.54, 4.16, 3.34, 3.68 | 10.89, 10.55, 9.07, 10.31, 11.36, 8.54 | -1.07, -1.12, -0.39, -0.67, -1.24, -0.77 |
| *Qtgw.acs-6B.2* | E8 | Xwmc341–Xbarc198 | 73 | 3.16 | 4.14 | 0.86 |
| *Qtgw.acs-7A* | E7 | Xbarc157–Xbarc23 | 37 | 4.01 | 5.60 | 0.85 |
| *Qtgw.acs-7B.1* | E1, E2, E5, E6, E7, BLUP | Xgwm569–Xbarc1073 | 4 | 4.00, 4.14, 5.20, 3.81, 4.12, 3.63 | 8.50, 9.22, 9.47, 8.55, 8.87, 9.95 | -0.76, -0.69, -0.95, -0.81, -0.71, -0.79 |
| *Qtgw.acs-7B.2* | E6 | Xbarc278–Xbarc32 | 50 | 3.02 | 2.84 | 0.52 |
| *Qtgw.acs-7B.3* | E8 | Xpsp3033–Xgwm302 | 55 | 3.20 | 4.25 | -0.87 |
| *Qtgw.acs-7B.4* | E2 | Xbarc258–Xmag353 | 74 | 5.24 | 8.62 | -1.72 |
| *Qtgw.acs-7D.1* | E1, E2, E4, E5, E7, E8, BLUP | Xgwm635–Xgwm428 | 6 | 5.40, 6.24, 6.38, 4.97, 3.01,5.94, 4.65 | 9,87, 12.64, 9.11, 10.65, 8.43, 10.07, 9.45 | 0.85, 1.22, 0.98, 1.23, 1.04, 1.01, 0.80 |
| *Qtgw.acs-7D.2* | E2, E4, E6 | Xcfd46–Xwmc438 | 21 | 3.12, 3.83, 4.04 | 6.13, 9.87, 10.24 | 1.24, 1.16, 0.89 |

RIL, recombinant inbred line; TGW, thousand grain weight; Site (cM), the most likely position of the putative QTL on the specific chromosome; LOD, logarithm of odds; BLUP, best linear unbiased prediction; PVE (%), the proportion of phenotypic variations explained by additive QTL; Add, the additive effect, of which a positive value indicates the Q9086 allele having positive effect on the trait, and negative value represents Longjian 19 allele having a positive effect. E1-E6, experimental environments at Yuzhong farm station in six years from 2013 to 2018, respectively; E7 and E8, experimental environments at Tongwei farm station in 2017 and 2018, respectively.

**SUPPLEMENTARY TABLE 3 |** Initial QTL information collected from the 45 individual mapping populations and references

| **ID** |  | **Parents** | **Type of population** | **Population size** | **Number of QTLs** | **Reference** |
| --- | --- | --- | --- | --- | --- | --- |
| 1 |  | Prinz×W-7984 | BC2F2 | 72 | 8 | Huang et al., 2003 |
| 2 |  | RL4452×AC Domain | DH | 182 | 6 | McCartney et al., 2005 |
| 3 |  | AC Karma×87E03-S2B1 | DH | 414 | 6 | Huang et al., 2006 |
| 4 |  | Superb×BW278 | DH | 402 | 6 | Cuthbert et al., 2008 |
| 5 |  | Chara×WW2449 | DH | 190 | 4 | Raman et al., 2009 |
| 6 |  | Chuan35050×Shannong483 | RIL | 131 | 5 | Sun et al., 2009 |
| 7 |  | Yu8679×Heshangmai | RIL | 142 | 18 | Wang et al., 2009 |
| 8 |  | Rye Selection111×Chinese Spring | RIL | 185 | 9 | Ramya et al., 2010 |
| 9 |  | MN98550×MN99394 | RIL | 139 | 4 | Tsilo et al., 2010 |
| 10 |  | Line3228×Jing4839 | F2 | 237 | 9 | Wang et al., 2010 |
| 11 |  | 6044×01-35 | RIL | 187 | 3 | Chen et al., 2011 |
| 12 |  | LDN hereafter × G18-16 | RIL | 152 | 10 | Peleg et al., 2011 |
| 13 |  | Hanxuan10×Lumai14 | DH | 150 | 7 | Wu et al., 2011 |
| 14 |  | G18-16×Langdon | RIL | 152 | 2 | Yan et al., 2011 |
| 15 |  | Kukri×RAC875 | DH | 368 | 9 | Bennett et al., 2012 |
| 16 |  | Poland wheat×Zhong13 | RIL | 99 | 3 | Li et al., 2012 |
| 17 |  | Wichita×Cheyenne | RIL | 90 | 3 | Mengistu et al., 2012 |
| 18 |  | Rye Selection111×Chinese Spring | RIL | 92 | 8 | Mir et al., 2012 |
| 19 |  | PDW233×Bhalegaon 4 | RIL | 140 | 6 | Patil et al., 2012 |
| 20 |  | Rye Selection111×Chinese Spring | RIL | 185 | 2 | Prashant et al., 2012 |
| 21 |  | 6044×01-35 | RIL | 187 | 7 | Wang et al., 2012a |
| 22 |  | 01-35×6044 | RIL | 187 | 2 | Wang et al., 2012b |
| 23 |  | HTRI11712×HTRI105 | F2 | 133 | 11 | Zaynali Nezhad et al., 2011 |
| 24 |  | Nanda2419×Wangshuibai | RIL | 230 | 6 | Jia et al., 2013 |
| 25 |  | Jing411×Hongmangchun21 | RIL | 177 | 1 | Liu et al., 2013 |
| 26 |  | Keumkang×Olgeuru | DH | 122 | 3 | Lee et al., 2014 |
| 27 |  | WL711×C306 | RIL | 206 | 5 | Shukla et al., 2015 |
| 28 |  | SHW-L1×Chuanmai32 | RIL | 171 | 7 | Yu et al., 2014 |
| 29 |  | Shi4185×Fu4185 | F2 | 249 | 2 | Cheng et al., 2015 |
| 30 |  | Longjian19×Q9086 | RIL | 120 | 15 | Hu et al., 2015 |
| 31 |  | Line0911-46×Line42 | F2 | 210 | 4 | Li et al., 2015 |
| 32 |  | Yanda1817×Beinong6 | RIL | 269 | 26 | Wu et al., 2015 |
| 33 |  | HD2808×HUW510 | RIL | 397 | 2 | Bhusal et al., 2017 |
| 34 |  | Pubing3504×Jing4839. | F2 | 282 | 7 | Chen et al., 2017 |
| 35 |  | Huapei3 × Yumai57 | DH | 168 | 12 | Deng et al., 2017 |
| 36 |  | Excalibur×Kukri | DH | 192 | 7 | Gahlaut et al., 2017 |
| 37 |  | WH542×synthetic derivative | RIL | 286 | 5 | Krishnappa et al., 2017 |
| 38 |  | UC1113×Kofa | RIL | 93 | 12 | Roncallo et al., 2017 |
| 39 |  | Annong0711×Yannong19 | BC1F2 | 680 | 2 | Wang et al., 2017 |
| 40 |  | ND3338×JD6 | DH | 203 | 49 | Guan et al., 2018 |
| 41 |  | NW1014×HUW468 | RIL | 106 | 1 | Kumari et al., 2018 |
| 42 |  | Shixin828×Kenong2007 | RIL | 163 | 17 | Su et al., 2018 |
| 43 |  | WL711×C306 | RIL | 206 | 5 | Goel et al., 2019 |
| 44 |  | Huapei3×Yumai57 | DH | 168 | 9 | Zhang et al., 2019 |
| 45 |  | Dalibao×BYL8 | RIL | 547 | 4 | Xin et al., 2020 |

RIL, recombinant inbred line populations; DH, double haploid populations

**REFERENCES**

Bennett, D., Izanloo, A., Reynolds, M., Kuchel, H., Langridge, P., Schnurbusch, T. (2012). Genetic dissection of grain yield and physical grain quality in bread wheat (*Triticum aestivum* L.) under water-limited environments. *Theor. Appl. Genet.* 125, 255-271. doi:10.1007/s00122-012-1831-9

Bhusal, N., Sarial, A. K., Sharma, P., Sareen, S. (2017). Mapping QTLs for grain yield components in wheat under heat stress. *PLoS One* 12, e0189594. doi:10.1371/journal.pone.0189594

Chen, D., Wu, X., Wu, K., Zhang, J., Liu, W., Yang, X., et al. (2017). Novel and favorable genomic regions for spike related traits in a wheat germplasm Pubing 3504 with high grain number per spike under varying environments. *J. Integr. Agr.* 16, 2386-2401. doi:10.1016/S2095-3119(17)61711-8

Chen, J. H., Lan, J. H., Wang, H., Wang, W. W., Tian, J. C. (2011). QTL mapping for traits of kernel morphology and grain weight in common wheat. *J. Triticeae Crops* 31, 1001-1006. doi:10.7606/j.issn.1009-1041.2011.06.001

Cheng, X. J., Chai, L. L., Chen, Z. Y., Xu, L., Zhai, H. J., Zhao, A. J., et al. (2015). Identification and characterization of a high kernel weight mutant induced by gamma radiation in wheat (*Triticum aestivum* L.). *BMC Genet.* 16, 127. doi:10.1186/s12863-015-0285-x

Cuthbert, J. L., Somers, D. J., Brûlé-Babel, A. L., Brown, P. D., Crow, G. H. (2008). Molecular mapping of quantitative trait loci for yield and yield components in spring wheat (*Triticum aestivum* L.). *Theor. Appl. Genet.* 117, 595-608. doi:10.1007/s00122-008-0804-5

Deng, Z., Cui, Y., Han, Q., Fang, W., Li, J., Tian, J. (2017). Discovery of consistent qtls of wheat spike-related traits under nitrogen treatment at different development stages. *Front. Plant Sci.* 8, 2120. doi:10.3389/fpls.2017.02120

Gahlaut, V., Jaiswal, V., Tyagi, B. S., Singh, G., Sareen, S., Balyan, H. S., et al. (2017). QTL mapping for nine drought-responsive agronomic traits in bread wheat under irrigated and rain-fed environments. *PLoS One* 12, e0182857. doi:10.1371/journal.pone.0182857

Goel, S., Singh, K., Singh, B., Grewal, S., Dwivedi, N., Alqarawi, A. A., et al. (2019). Analysis of genetic control and QTL mapping of essential wheat grain quality traits in a recombinant inbred population. *PLoS One* 14, e0200669. doi:10.1371/journal.pone.0200669

Guan, P., Lu, L., Jia, L., Kabir, M. R., Zhang, J., Lan, T., et al. (2018). Global QTL analysis identifies genomic regions on chromosomes 4A and 4B harboring stable loci for yield-related traits across different environments in wheat (*Triticum aestivum* L.). *Front. Plant Sci.* 9, 529. doi:10.3389/fpls.2018.00529

Hu, L. L., Ye, Y. Q., Lv, T. T., Li, M. F., Liu, Y., Chang, L., et al. (2015). QTL mapping and genetic analysis for grain weight (*Triticum aestivum*) under different water environments. *Acta Pratacul. Sin.* 8, 118-129. doi:10.11686/cyxb2015071

Huang, X. Q., Cloutier, S., Lycar, L., Radovanovic, N., Humphreys, D. G., Noll, J. S., et al. (2006). Molecular detection of QTLs for agronomic and quality traits in a doubled haploid population derived from two Canadian wheats (*Triticum aestivum* L.). *Theor. Appl. Genet.* 113, 753-766. doi:10.1007/s00122-006-0346-7

Huang, X. Q., Cöster, H., Ganal, M. W., Röder, M. S. (2003). Advanced backcross QTL analysis for the identification of quantitative trait loci alleles from wild relatives of wheat ( *Triticum aestivum* L.). *Theor. Appl. Genet.* 106, 1379-1389. doi:10.1007/s00122-002-1179-7

Jia, H., Wan, H., Yang, S., Zhang, Z., Kong, Z., Xue, S., et al. (2013). Genetic dissection of yield-related traits in a recombinant inbred line population created using a key breeding parent in China's wheat breeding. *Theor. Appl. Genet.* 126, 2123-2139. doi:10.1007/s00122-013-2123-8

Krishnappa, G., Singh, A. M., Chaudhary, S., Ahlawat, A. K., Singh, S. K., Shukla, R. B., et al. (2017). Molecular mapping of the grain iron and zinc concentration, protein content and thousand kernel weight in wheat (*Triticum aestivum* L.). *PLoS One* 12, e0174972. doi:10.1371/journal.pone.0174972

Kumari, S., Jaiswal, V., Mishra, V. K., Paliwal, R., Balyan, H. S., Gupta, P. K. (2018). QTL mapping for some grain traits in bread wheat (*Triticum aestivum* L.). *Physiol. Mol. Biol. Pla.* 24, 909-920. doi:10.1007/s12298-018-0552-1

Lee, H. S., Jung, J. U., Kang, C. S., Heo, H. Y., Park, C. S. (2014). Mapping of QTL for yield and its related traits in a doubled haploid population of Korean wheat. *Plant Biotechnol. Rep.* 8, 443-454. doi:10.1007/s11816-014-0337-0

Li, M. X., Wang, Z. L., Liang, Z. Y., Shen, W. N., Sun, F. L., Xi, Y. J., et al. (2015). Quantitative trait loci analysis for kernel-related characteristics in common wheat (*Triticum aestivum* L.). *Crop Sci.* 55, 1485-1493. doi:10.2135/cropsci2014.09.0616

Li, M. X., Yang, R., Li, Y. M., Cui, G. B., Wang, Z. L. (2012). QTL analysis of kernel characteristics using a recombinant inbred lines (RILs) population derived from the cross of *Tiriticum polonicum* L. and *Triticum aestivum* L. line "Zhong 13". *J Triticeae Crops* 32, 813-819. doi:10.7606/j.issn.1009-1041.2012.05.002

Liu, S. N., Gan, J. F., Zhang, H. P., Chang, C., Lu, J., Si, H. Q., et al. (2013). QTL mapping and analysis of correlation between chlorophyll content and 1000-kernal weight in RILs population of wheat. *J. Anhui Agric. Univ.* 40, 570-574. doi:10.13610/j.cnki.1672-352x.2013.04.017

McCartney, C. A., Somers, D. J., Humphreys, D. G., Lukow, O., Ames, N., Noll, J., et al. (2005). Mapping quantitative trait loci controlling agronomic traits in the spring wheat cross RL4452x'AC Domain'. *Genome* 48, 870-883. doi:10.1139/g05-055

Mengistu, N., Baenziger, P. S., Eskridge, K. M., Dweikat, I., Wegulo, S. N., Gill, K. S., et al. (2012). Validation of QTL for grain yield-related traits on wheat chromosome 3A using recombinant inbred chromosome lines. *Crop Sci.* 52, 1622-1632. doi:10.2135/cropsci2011.12.0677

Mir, R. R., Kumar, N., Jaiswal, V., Girdharwal, N., Prasad, M., Balyan, H. S., et al. (2012). Genetic dissection of grain weight in bread wheat through quantitative trait locus interval and association mapping. *Mol. Breeding* 29, 963-972. doi:10.1007/s11032-011-9693-4

Patil, R. M., Tamhankar, S. A., Oak, M. D., Raut, A. L., Honrao, B. K., Rao, V. S., et al. (2012). Mapping of QTL for agronomic traits and kernel characters in durum wheat (*Triticum durum* Desf.). *Euphytica* 190, 117-129. doi:10.1007/s10681-012-0785-y

Peleg, Z., Fahima, T., Korol, A. B., Abbo, S., Saranga, Y. (2011). Genetic analysis of wheat domestication and evolution under domestication. *J. Exp. Bot.* 62, 5051-5061. doi:10.1093/jxb/err206

Prashant, R., Kadoo, N., Desale, C., Kore, P., Dhaliwal, H. S., Chhuneja, P., et al. (2012). Kernel morphometric traits in hexaploid wheat (*Triticum aestivum* L.) are modulated by intricate QTL × QTL and genotype × environment interactions. *J. Cereal Sci.* 56, 432-439. doi:10.1016/j.jcs.2012.05.010

Raman, R., Allen, H., Diffey, S., Raman, H., Martin, P., McKelvie, K. (2009). Localisation of quantitative trait loci for quality attributes in a doubled haploid population of wheat (*Triticum aestivum* L.). *Genome* 52, 701-715. doi:10.1139/g09-045

Ramya, P., Chaubal, A., Kulkarni, K., Gupta, L., Kadoo, N., Dhaliwal, H. S., et al. (2010). QTL mapping of 1000-kernel weight, kernel length, and kernel width in bread wheat (*Triticum aestivum* L.). *J. Appl. Genet.* 51, 421-429. doi:10.1007/bf03208872

Roncallo, P. F., Akkiraju, P. C., Cervigni, G. L., Echenique, V. C. (2017). QTL mapping and analysis of epistatic interactions for grain yield and yield-related traits in *Triticum turgidum* L. var. *durum*. *Euphytica* 213, 277. doi:10.1007/s10681-017-2058-2

Shukla, S., Singh, K., Patil, R. V., Kadam, S., Bharti, S., Prasad, P., et al. (2015). Genomic regions associated with grain yield under drought stress in wheat (*Triticum aestivum* L.). *Euphytica* 203, 449-467. doi:10.1007/s10681-014-1314-y

Su, Q., Zhang, X., Zhang, W., Zhang, N., Song, L., Liu, L., et al. (2018). QTL detection for kernel size and weight in bread wheat (*Triticum aestivum* L.) using a high-density SNP and SSR-based linkage map. *Front. Plant Sci.* 9, 1484. doi:10.3389/fpls.2018.01484

Sun, X. Y., Ke, W., Zhao, Y., Kong, F. M., Han, G. Z., Jiang, H. M., et al. (2009). QTL analysis of kernel shape and weight using recombinant inbred lines in wheat. *Euphytica* 165, 615. doi:10.1007/s10681-008-9794-2

Tsilo, T. J., Hareland, G. A., Simsek, S., Chao, S., Anderson, J. A. (2010). Genome mapping of kernel characteristics in hard red spring wheat breeding lines. *Theor. Appl. Genet.* 121, 717-730. doi:10.1007/s00122-010-1343-4

Wang, H., Lan, J. H., Tian, J. C. (2012a). Dynamic QTL analysis of kernel weight in wheat at different developmental stages. *J. Plant Genet. Resour.* 13, 1055-1060. doi:10.3969/j.issn.1672-1810.2012.06.022

Wang, J. S., Liu, W. H., Wang, H., Li, L. H., Wu, J., Yang, X. M., et al. (2010). QTL mapping of yield-related traits in the wheat germplasm 3228. *Euphytica* 177, 277-292. doi:10.1007/s10681-010-0267-z

Wang, R. X., Zhang, X. Y., Wu, L., Wang, R., Hai, L., You, G. X., et al. (2009). QTL analysis of grain size and related traits in winter wheat under different ecological environments. *Sci. Agric. Sin.* 42, 398-407. doi:10.3864/j.issn.0578-1752.2009.02.003

Wang, S. X., Niu, Y., Chen, C. L., Zheng, L., Ma, H. H., Shi, M. L., et al. (2017). Genome-wide QTL analysis for wheat grain yield per plant and other related traits. *J. Anhui Agric. Univ.* 44, 720-725. doi:10.13610/j.cnki.1672-352x.20170811.026

Wang, W. W., Lan, J. H., Tian, J. C. (2012b). The preliminary study on qtl of grain filling rate and grain weight in wheat. *Chin. Agric. Sci. Bull.* 28, 63-70. doi:10.3969/j.issn.1000-6850.2012.36.010

Wu, Q., Chen, Y., Zhou, S., Fu, L., Chen, J., Xiao, Y., et al. (2015). High-density genetic linkage map construction and QTL mapping of grain shape and size in the wheat population Yanda1817 × Beinong6. *PLoS One* 10, e0118144. doi:10.1371/journal.pone.0118144

Wu, X., Chang, X., Jing, R. (2011). Genetic analysis of carbon isotope discrimination and its relation to yield in a wheat doubled haploid population. *J. Integr. Plant Biol.* 53, 719-730. doi:10.1111/j.1744-7909.2011.01067.x

Xin, F., Zhu, T., Wei, S. W., Han, Y. C., Zhao, Y., Zhang, D. Z., et al. (2020). QTL mapping of kernel traits and validation of a major QTL for kernel length-width ratio using SNP and bulked segregant analysis in wheat. *Sci. Rep.* 10, 25. doi:10.1038/s41598-019-56979-7

Yan, J., Zhang, L. L., Wang, X. M., Xue, W. T., Yang, R. Z., T.Fahima, et al. (2011). QTL mapping of yield-related traits in durum wheat × wild emmer wheat RIL population. *J. Shandong Agric. Univ. (Nat. Sci.)* 42, 163-171. doi:10.3969/j.issn.1000-2324.2011.02.001

Yu, M., Chen, G. Y., Zhang, L. Q., Liu, Y. X., Liu, D. C., Wang, J., et al. (2014). QTL mapping for important agronomic traits in synthetic hexaploid wheat derived from *Aegiliops tauschii ssp. tauschi*i. *J. Integr. Agr.* 13, 1835-1844. doi:10.1016/S2095-3119(13)60655-3

Zaynali Nezhad, K., Weber, W. E., Röder, M. S., Sharma, S., Lohwasser, U., Meyer, R. C., et al. (2011). QTL analysis for thousand-grain weight under terminal drought stress in bread wheat (*Triticum aestivum* L.). *Euphytica* 186, 127-138. doi:10.1007/s10681-011-0559-y

Zhang, H., Sun, J., Chen, J., Liu, B., Deng, Z., Li, R., et al. (2019). Conditional QTL analysis of three yield components in wheat (*Triticum aestivum* L.). *J. Triticeae Crops* 39, 42-49. doi:10.7606/j.issn.1009-1041.2019.01.06

**SUPPLEMENTARY TABLE 4 |** Description of 67 MQTLs identified for TGW

| **MQTL** | **Position** | **95% CI (cM)** | **Flanking marker** | **Physical interval** | **Physical distance (Mb)** | **QTL number** | **Homology‑based candidate gene** |
| --- | --- | --- | --- | --- | --- | --- | --- |
| MQTL-1A-1 | 41.39 | 0.94 | BS00022870_51-IAAV8824 | 48588559-121046541 | 72.46 | 3 | TraesCS1A02G083100 |
|  |  |  |  |  |  |  | TraesCS1A02G086500 |
|  |  |  |  |  |  |  | TraesCS1A02G113400 |
| MQTL-1A-2 | 45.91 | 0.20 | Xwmc611-Xgpw2045 | 152920754-238257622 | 85.34 | 6 | TraesCS1A02G136500 |
| MQTL-1A-3 | 47.97 | 0.89 | NA |  | NA | 3 |  |
| MQTL-1A-4 | 107.04 | 3.37 | BobWhite_c1027_1127-BS00067741_51 | 586993772-590003251 | 3.01 | 3 |  |
| MQTL-1B-1 | 47.10 | 1.95 | Xwmc728-BS00084990_51 | 479614334-673745712 | 194.13 | 5 | TraesCS1B02G293100 |
|  |  |  |  |  |  |  | TraesCS1B02G449700 |
|  |  |  |  |  |  |  | TraesCS1B02G288100 |
|  |  |  |  |  |  |  | TraesCS1B02G352200 |
|  |  |  |  |  |  |  | TraesCS1B02G274300 |
|  |  |  |  |  |  |  | TraesCS1B02G341000 |
|  |  |  |  |  |  |  | TraesCS1B02G354000 |
| MQTL-1B-2 | 63.50 | 1.55 | BS00069723_51-wsnp_Ra_c12888_20519578 | 456253786-471193698 | 14.94 | 6 |  |
| MQTL-1B-3 | 71.98 | 0.84 | Xwmc230-wPt-3282 | 42183239-75079607 | 32.9 | 7 | TraesCS1B02G081100 |
| MQTL-1B-4 | 73.13 | 0.47 | wPt-2230-XksuE18 | 6225015-34953057 | 28.73 | 2 | TraesCS1B02G032400 |
| MQTL-1B-5 | 77.16 | 2.51 | Xbarc120-Xwmc213 | 26399646-155507732 | 129.11 | 2 | TraesCS1B02G104900 |
|  |  |  |  |  |  |  | TraesCS1B02G142300 |
|  |  |  |  |  |  |  | TraesCS1B02G142100 |
|  |  |  |  |  |  |  | TraesCS1B02G142200 |
|  |  |  |  |  |  |  | TraesCS1B02G058600 |
| MQTL-1B-6 | 86.03 | 0.29 | Xwmc85-wsnp_BE637971B_Ta_1_20 | 8816341-10204938 | 1.39 | 5 |  |
| MQTL-2A-1 | 6.08 | 5.22 | NA |  | NA | 2 |  |
| MQTL-2A-2 | 13.19 | 1.69 | BobWhite_c19433_329-wPt-2120 | 30866076-62315257 | 31.45 | 2 | TraesCS2A02G099900 |
| MQTL-2A-3 | 20.41 | 3.05 | Xbarc1138-Xcmwg682 | 2471848-3965433 | 1.49 | 6 |  |
| MQTL-2A-4 | 22.71 | 0.10 | wsnp_Ex_c13260_20914585-BS00022896_51 | 509548533-612845080 | 103.3 | 6 | TraesCS2A02G331800 |
|  |  |  |  |  |  |  | TraesCS2A02G336000 |
|  |  |  |  |  |  |  | TraesCS2A02G312200 |
|  |  |  |  |  |  |  | TraesCS2A02G306200 |
| MQTL-2A-5 | 28.72 | 2.55 | wPt-3244-Kukri_c23400_362 | 709712323-765710294 | 56 | 6 | TraesCS2A02G517100 |
|  |  |  |  |  |  |  | TraesCS2A02G464000 |
|  |  |  |  |  |  |  | TraesCS2A02G548100 |
| MQTL-2A-6 | 45.24 | 0.21 | wPt-8427-Tdurum_contig25539_248 | 672526565-758287054 | 62.99 | 7 | TraesCS2A02G423500 |
|  |  |  |  |  |  |  | TraesCS2A02G424600 |
|  |  |  |  |  |  |  | TraesCS2A02G464000 |
|  |  |  |  |  |  |  | TraesCS2A02G517100 |
|  |  |  |  |  |  |  | TraesCS2A02G548100 |
| MQTL-2B-1 | 137.11 | 6.24 | Xgwm148-wsnp_Ex_c9935_16358536 | 108458841-174345668 | 65.89 | 2 | TraesCS2B02G152900 |
| MQTL-2B-2 | 146.47 | 4.79 | Excalibur_c25234_143-Xcdo665 | 66214720-69499989 | 3.29 | 8 |  |
| MQTL-2B-3 | 172.52 | 0.04 | BS00075852_51-Xwmc213 | 46891603-302332067 | 255.44 | 5 | TraesCS2B02G211100 |
|  |  |  |  |  |  |  | TraesCS2B02G219300 |
|  |  |  |  |  |  |  | TraesCS2B02G136100 |
|  |  |  |  |  |  |  | TraesCS2B02G116900 |
|  |  |  |  |  |  |  | TraesCS2B02G201900 |
|  |  |  |  |  |  |  | TraesCS2B02G152900 |
| MQTL-2D-1 | 49.32 | 0.79 | Kukri_c32234_413-IACX6231 | 541473362-577126804 | 35.65 | 5 | TraesCS2D02G464900 |
| MQTL-2D-2 | 86.57 | 0.38 | Kukri_c365_345-Excalibur_c73791_215 | 635950371-645934113 | 9.98 | 7 |  |
| MQTL-3A-1 | 47.93 | 0.77 | Tdurum_contig12371_132-wsnp_Ku_c44089_51445136 | 47825432-95021415 | 47.2 | 9 | TraesCS3A02G103800 |
|  |  |  |  |  |  |  | TraesCS3A02G105400 |
|  |  |  |  |  |  |  | TraesCS3A02G108100 |
|  |  |  |  |  |  |  | TraesCS3A02G077900 |
| MQTL-3A-2 | 52.40 | 2.24 | Xgwm155-TC77302 | 702961806-739640504 | 36.68 | 2 |  |
| MQTL-3A-3 | 86.67 | 8.89 | Xbarc51-Xbarc1177 | 716795032-746581359 | 29.79 | 3 |  |
| MQTL-3B-1 | 39.76 | 5.39 | wsnp_CAP11_c232_211960-wsnp_Ra_c18153_27161629 | 20841477-26649466 | 5.81 | 3 |  |
| MQTL-3B-2 | 49.67 | 0.66 | wPt-5064-RAC875_c5799_170 | 17900156-31813747 | 13.91 | 5 |  |
| MQTL-3B-3 | 53.84 | 3.29 | wsnp_Ex_rep_c68193_66971396-IAAV6566 | 730286103-736712583 | 6.43 | 2 |  |
| MQTL-3B-4 | 61.20 | 3.38 | wPt-4719-wsnp_JD_c4413_5541190 | 724626731-742171244 | 17.54 | 4 |  |
| MQTL-3B-5 | 82.10 | 7.80 | RAC875_c37741_476-BS00068415_51 | 820501695-826082809 | 5.58 | 2 |  |
| MQTL-3D-1 | 108.09 | 6.43 | Excalibur_c11824_103-wsnp_Ex_c8409_14170476 | 4486783-24606257 | 20.12 | 3 |  |
| MQTL-3D-2 | 124.90 | 17.27 | Xbcd361-Xbarc270 | 551998447-584711053 | 32.71 | 4 |  |
| MQTL-3D-3 | 146.89 | 30.76 | BS00004334_51-Xgpw307 | 574773288-615452957 | 40.48 | 2 |  |
| MQTL-4A-1 | 17.03 | 4.08 | wsnp_BM138178A_Ta_2_1-RAC875_rep_c69241_454 | 37813853-102387645 | 64.57 | 2 | TraesCS4A02G074700 |
|  |  |  |  |  |  |  | TraesCS4A02G090600 |
|  |  |  |  |  |  |  | TraesCS4A02G051600 |
|  |  |  |  |  |  |  | TraesCS4A02G047100 |
|  |  |  |  |  |  |  | TraesCS4A02G074300 |
|  |  |  |  |  |  |  | TraesCS4A02G059200 |
|  |  |  |  |  |  |  |  |
| MQTL-4A-2 | 28.26 | 3.16 | BS00012146_51-wsnp_Ra_c1022_2067517 | 592546581-595986207 | 3.44 | 4 | TraesCS4A02G294000 |
| MQTL-4A-3 | 41.73 | 7.83 | BS00021727_51-RAC875_c20429_903 | 606733202-617948862 | 11.22 | 3 |  |
| MQTL-4A-4 | 52.29 | 1.31 | wsnp_Ex_c3988_7221220-Excalibur_c11968_204 | 666149052-679274348 | 13.13 | 7 |  |
| MQTL-4A-5 | 65.27 | 2.32 | BS00021957_51-BobWhite_c8680_918 | 693278307-705179194 | 11.9 | 4 |  |
| MQTL-5B-1 | 35.19 | 3.35 | Kukri_c59657_805-BS00090411_51 | 690091689-700330427 | 10.24 | 3 | TraesCS5B02G538300 |
| MQTL-5B-2 | 46.65 | 8.49 | wsnp_Ex_c607_1204733-Xgwm191 | 10438371-80197929 | 69.76 | 2 |  |
| MQTL-5B-3 | 57.34 | 2.82 | BobWhite_rep_c50362_537-BS00022956_51 | 510885167-653433467 | 142.55 | 4 | TraesCS5B02G375800 |
|  |  |  |  |  |  |  | TraesCS5B02G400000 |
|  |  |  |  |  |  |  | TraesCS5B02G381300 |
|  |  |  |  |  |  |  | TraesCS5B02G399200 |
|  |  |  |  |  |  |  | TraesCS5B02G378600 |
| MQTL-5B-4 | 62.21 | 2.12 | Xgwm213-BS00022899_51 | 418808253-652477863 | 233.67 | 5 | TraesCS5B02G241200 |
|  |  |  |  |  |  |  | TraesCS5B02G265600 |
|  |  |  |  |  |  |  | TraesCS5B02G286000 |
|  |  |  |  |  |  |  | TraesCS5B02G292100 |
|  |  |  |  |  |  |  | TraesCS5B02G307600 |
|  |  |  |  |  |  |  | TraesCS5B02G375800 |
|  |  |  |  |  |  |  | TraesCS5B02G378600 |
|  |  |  |  |  |  |  | TraesCS5B02G381300 |
|  |  |  |  |  |  |  | TraesCS5B02G399200 |
|  |  |  |  |  |  |  | TraesCS5B02G400000 |
| MQTL-5B-5 | 80.53 | 1.22 | wsnp_Ex_rep_c68017_66762485-BS00037487_51 | 498886408-601419385 | 102.53 | 6 | TraesCS5B02G375800 |
|  |  |  |  |  |  |  | TraesCS5B02G378600 |
|  |  |  |  |  |  |  | TraesCS5B02G381300 |
|  |  |  |  |  |  |  | TraesCS5B02G399200 |
|  |  |  |  |  |  |  | TraesCS5B02G400000 |
| MQTL-5D-1 | 77.22 | 3.14 | Xcfd156-Xgwm212 | 464108642-472630290 | 8.52 | 6 | TraesCS5D02G404500 |
|  |  |  |  |  |  |  | TraesCS5D02G404000 |
| MQTL-5D-2 | 130.59 | 1.30 | BS00066615_51-wsnp_Ex_c13223_20866191 | 16387006-17911254 | 1.52 | 2 |  |
| MQTL-6A-1 | 126.64 | 4.05 | Kukri_c93507_110-wsnp_Ex_c9763_16125630 | 11158952-16566043 | 5.41 | 5 |  |
| MQTL-6A-2 | 144.43 | 1.53 | wPt-2153-wPt-8266 | 7300027-13439851 | 6.14 | 3 |  |
| MQTL-6A-3 | 152.01 | 0.10 | BS00065082_51-wsnp_Ex_c965_1845676 | 558922282-581747079 | 22.82 | 4 | TraesCS6A02G335900 |
| MQTL-6A-4 | 157.03 | 0.59 | Xbarc3-wsnp_Ku_c7794_13356946 | 85284828-87651066 | 2.37 | 5 |  |
| MQTL-6A-5 | 168.72 | 3.95 | RAC875_rep_c72327_349-IAAV5585 | 522610130-570378542 | 47.77 | 3 | TraesCS6A02G306200 |
|  |  |  |  |  |  |  | TraesCS6A02G309900 |
|  |  |  |  |  |  |  | TraesCS6A02G321000 |
|  |  |  |  |  |  |  | TraesCS6A02G335900 |
|  |  |  |  |  |  |  | TraesCS6A02G321400 |
| MQTL-6A-6 | 189.61 | 2.16 | CAP8_c1361_367-BS00063109_51 | 535144191-593007474 | 57.86 | 4 | TraesCS6A02G306200 |
|  |  |  |  |  |  |  | TraesCS6A02G309900 |
|  |  |  |  |  |  |  | TraesCS6A02G321000 |
|  |  |  |  |  |  |  | TraesCS6A02G321400 |
|  |  |  |  |  |  |  | TraesCS6A02G335900 |
| MQTL-6A-7 | 207.13 | 0.72 | Xabc175-BS00011578_51 | 520760696-611485398 | 90.72 | 2 | TraesCS6A02G306200 |
|  |  |  |  |  |  |  | TraesCS6A02G309900 |
|  |  |  |  |  |  |  | TraesCS6A02G321000 |
|  |  |  |  |  |  |  | TraesCS6A02G321400 |
|  |  |  |  |  |  |  | TraesCS6A02G335900 |
|  |  |  |  |  |  |  | TraesCS6A02G389100 |
|  |  |  |  |  |  |  | TraesCS6A02G377300 |
| MQTL-6B-1 | 65.26 | 1.38 | BS00022155_51-Excalibur_c26172_359 | 469787969-470914665 | 1.13 | 5 |  |
| MQTL-6B-2 | 71.22 | 1.51 | wsnp_Ku_rep_c72013_71735741-BS00065962_51 | 640973852-643632254 | 2.66 | 4 |  |
| MQTL-6B-3 | 76.07 | 0.40 | BS00064620_51-TA002465-0455-w | 49420085-115700997 | 66.28 | 4 |  |
| MQTL-6B-4 | 84.66 | 1.81 | wsnp_Ex_c1383_2652398-BS00036830_51 | 681317537-686688263 | 5.37 | 7 |  |
| MQTL-6B-5 | 107.59 | 1.13 | wPt-6967-wPt-5885 | 603797787-713122921 | 109.333 | 2 | TraesCS6B02G379500 |
|  |  |  |  |  |  |  | TraesCS6B02G352100 |
|  |  |  |  |  |  |  | TraesCS6B02G366700 |
|  |  |  |  |  |  |  | TraesCS6B02G414700 |
|  |  |  |  |  |  |  | TraesCS6B02G351700 |
|  |  |  |  |  |  |  | TraesCS6B02G430100 |
|  |  |  |  |  |  |  | TraesCS6B02G421600 |
| MQTL-7A-1 | 171.17 | 14.30 | Tdurum_contig4885_1536-RAC875_c3450_836 | 68049419-78433665 | 10.38 | 2 | TraesCS7A02G111300 |
| MQTL-7A-2 | 204.22 | 0.94 | Xfba204-BS00090785_51 | 430866118-689920727 | 259.05 | 3 | TraesCS7A02G308300 |
|  |  |  |  |  |  |  | TraesCS7A02G312800 |
|  |  |  |  |  |  |  | TraesCS7A02G317400 |
|  |  |  |  |  |  |  | TraesCS7A02G416400 |
|  |  |  |  |  |  |  | TraesCS7A02G427500 |
|  |  |  |  |  |  |  | TraesCS7A02G466900 |
|  |  |  |  |  |  |  | TraesCS7A02G479100 |
| MQTL-7A-3 | 207.22 | 1.01 | wsnp_Ex_rep_c104560_89241494-wPt-3393 | 531193128-625428997 | 94.24 | 4 | TraesCS7A02G416400 |
|  |  |  |  |  |  |  | TraesCS7A02G427500 |
| MQTL-7A-4 | 210.78 | 1.36 | JD_c149_3175-wsnp_Ex_c17899_26666328 | 670767383-680341517 | 9.57 | 6 | TraesCS7A02G479100 |
| MQTL-7A-5 | 217.02 | 2.22 | Xmag828-Excalibur_c61603_1052 | 699296744-700705500 | 1.41 | 4 |  |
| MQTL-7A-6 | 222.59 | 1.58 | IAAV6957-BS00098482_51 | 675231796-698687620 | 23.46 | 2 | TraesCS7A02G506400 |
| MQTL-7B-1 | 56.73 | 1.70 | Xwmc376-wPt-4743 | 637640202-719825356 | 82.19 | 4 | TraesCS7B02G381500 |
| MQTL-7B-2 | 63.88 | 1.01 | Excalibur_c12499_2075-wPt-9925 | 643354784-650966859 | 7.61 | 8 | TraesCS7B02G381500 |
| MQTL-7B-3 | 72.49 | 1.50 | Xfba301-Xbarc278 | 430866118-595079803 | 164.21 | 3 | TraesCS7B02G316600 |
|  |  |  |  |  |  |  | TraesCS7B02G327600 |
| MQTL-7B-4 | 75.51 | 1.90 | wPt-4297-Kukri_c46447_1738 | 701641003-718359215 | 16.72 | 2 |  |
| MQTL-7B-5 | 81.35 | 0.16 | Tdurum_contig46338_2305-wPt-8936 | 737101514-741001569 | 3.9 | 3 |  |

NA, MQTL was not well matched the physical position on the wheat genome reference sequence of Chinese Spring.

**SUPPLEMENTARY TABLE 5 |** The information of 513 genes were identified in five core MQTL intervals

| **MQTL** | **Gene** | **Gene function annotation** | **MQTL** | **Gene** | **Gene function annotation** |
| --- | --- | --- | --- | --- | --- |
| MQTL-1B-6 | TraesCS1B02G018300 | Serine/threonine-protein kinase | MQTL-2D-2 | TraesCS2D02G589000 | 3-isopropylmalate dehydrogenase |
|  | TraesCS1B02G018500 | Serine/threonine-protein kinase |  | TraesCS2D02G589100 | UPF0235 protein |
|  | TraesCS1B02G018600 | Serine/threonine-protein kinase |  | TraesCS2D02G589200 | Cytochrome P450 family protein, expressed |
|  | TraesCS1B02G018700 | 12-oxophytodienoate reductase-like protein |  | TraesCS2D02G589300 | Glutathione S-transferase |
|  | TraesCS1B02G018800 | 12-oxophytodienoate reductase-like protein |  | TraesCS2D02G589400 | Glutathione S-transferase |
|  | TraesCS1B02G018900 | Ras-related protein |  | TraesCS3B02G036500 | Patatin |
|  | TraesCS1B02G019000 | Disease resistance protein (NBS-LRR class) family | | TraesCS3B02G036600 | Dihydroflavonol-4-reductase |
|  | TraesCS1B02G019100 | Ras-like protein |  | TraesCS3B02G036700 | Bowman-Birk type trypsin inhibitor |
|  | TraesCS1B02G019200 | Tubulin-specific chaperone cofactor E-like protein | MQTL-3B-2 | TraesCS3B02G036800 | Patatin |
|  | TraesCS1B02G019300 | Chaperone protein dnaJ |  | TraesCS3B02G036900 | Wound-induced protease inhibitor |
|  | TraesCS1B02G019400 | Glutathione S-transferase |  | TraesCS3B02G037000 | Trypsin inhibitor |
|  | TraesCS1B02G019500 | Serine/threonine-protein kinase |  | TraesCS3B02G037100 | Trypsin inhibitor |
|  | TraesCS1B02G019600 | 12-oxophytodienoate reductase-like protein |  | TraesCS3B02G037200 | Trypsin inhibitor |
|  | TraesCS1B02G019700 | 12-oxophytodienoate reductase-like protein |  | TraesCS3B02G037300 | Trypsin inhibitor |
|  | TraesCS1B02G019800 | 12-oxophytodienoate reductase-like protein |  | TraesCS3B02G037400 | Trypsin inhibitor |
|  | TraesCS1B02G019900 | Serine/threonine-protein kinase |  | TraesCS3B02G037500 | Disease resistance protein RPM1 |
|  | TraesCS1B02G020000 | Cysteine proteinase |  | TraesCS3B02G037600 | Bowman-Birk type trypsin inhibitor |
|  | TraesCS1B02G020100 | F-box family protein |  | TraesCS3B02G037700 | Ripening-related protein |
|  | TraesCS1B02G020200 | Protein kinase family protein |  | TraesCS3B02G037800 | Werner Syndrome-like exonuclease |
|  | TraesCS1B02G020300 | Nbs-lrr resistance protein |  | TraesCS3B02G037900 | Ripening-related protein |
|  | TraesCS1B02G020400 | NBS-LRR disease resistance protein | | TraesCS3B02G038000 | Ripening-related protein |
|  | TraesCS1B02G020500 | Wall-associated receptor kinase-like protein |  | TraesCS3B02G038100 | Kiwellin |
|  | TraesCS1B02G020600 | Receptor-like kinase |  | TraesCS3B02G038200 | Ripening-related protein |
|  | TraesCS1B02G020700 | Receptor kinase |  | TraesCS3B02G038300 | Trypsin inhibitor |
|  | TraesCS1B02G020800 | Transmembrane protein, putative (DUF594) |  | TraesCS3B02G038400 | Bowman-Birk type trypsin inhibitor |
|  | TraesCS1B02G020900 | NBS-LRR disease resistance protein-like protein | | TraesCS3B02G038500 | Pathogen-related protein |
|  | TraesCS1B02G021000 | O-methyltransferase-like protein |  | TraesCS3B02G038600 | Trypsin inhibitor |
|  | TraesCS1B02G021100 | Glutathione S-transferase |  | TraesCS3B02G038700 | Trypsin inhibitor |
|  | TraesCS1B02G021200 | RNA-binding family protein |  | TraesCS3B02G038800 | NADP dependent sorbitol 6-phosphate dehydrogenase family protein |
|  | TraesCS1B02G021300 | Phosphatidate cytidylyltransferase |  | TraesCS3B02G038900 | Protein kinase |
|  | TraesCS1B02G021400 | Glutathione S-transferase |  | TraesCS3B02G039000 | Mannose-6-phosphate isomerase |
|  | TraesCS1B02G021500 | Disease resistance protein |  | TraesCS3B02G039100 | MYB transcription factor |
|  | TraesCS1B02G021600 | Protein kinase family protein |  | TraesCS3B02G039200 | NBS-LRR disease resistance protein |
|  | TraesCS1B02G021700 | Transmembrane protein, putative (DUF247) |  | TraesCS3B02G039300 | Protein DETOXIFICATION |
|  | TraesCS1B02G021800 | Protein kinase family protein |  | TraesCS3B02G039400 | ARM repeat superfamily protein |
|  | TraesCS1B02G021900 | NBS-LRR disease resistance protein, putative, expressed | | TraesCS3B02G039500 | Nuclease S1 |
|  | TraesCS1B02G022000 | Serine/threonine-protein kinase |  | TraesCS3B02G039600 | Nuclease S1 |
|  | TraesCS1B02G022100 | NBS-LRR disease resistance protein-like protein | | TraesCS3B02G039700 | Nuclease S1 |
|  | TraesCS1B02G022200 | NBS-LRR disease resistance protein-like protein | | TraesCS3B02G039800 | Abscisic acid-deficient 4 |
|  | TraesCS1B02G022300 | Receptor-like kinase |  | TraesCS3B02G039900 | Transmembrane protein 214 |
|  | TraesCS1B02G022400 | Receptor-like kinase protein |  | TraesCS3B02G040000 | C2 domain containing protein |
|  | TraesCS1B02G022500 | Protein trichome birefringence |  | TraesCS3B02G040100 | NBS-LRR disease resistance protein, putative, expressed |
|  | TraesCS1B02G022600 | Transmembrane protein, putative (DUF594) |  | TraesCS3B02G040200 | Zinc finger family protein |
|  | TraesCS1B02G022700 | Zinc finger protein |  | TraesCS3B02G040300 | DUF1666 family protein |
|  | TraesCS1B02G022800 | V-type proton ATPase proteolipid subunit |  | TraesCS3B02G040400 | 30S ribosomal protein S10 |
|  | TraesCS1B02G022900 | Nuclear inhibitor of protein phosphatase 1 |  | TraesCS3B02G040500 | Cytochrome b6-f complex subunit 5 |
| MQTL-2D-2 | TraesCS2D02G567600 | Magnesium transporter, putative (DUF803) |  | TraesCS3B02G040600 | DNA-binding storekeeper protein-related transcriptional regulator |
|  | TraesCS2D02G567700 | MYB-related transcription factor |  | TraesCS3B02G040700 | DNA topoisomerase |
|  | TraesCS2D02G567800 | Disease resistance protein RPM1 |  | TraesCS3B02G040800 | Protein NEGATIVE REGULATOR OF RESISTANCE |
|  | TraesCS2D02G567900 | Tetratricopeptide repeat (TPR)-like superfamily protein | | TraesCS3B02G040900 | Metal tolerance protein |
|  | TraesCS2D02G568000 | NAC domain protein |  | TraesCS3B02G041000 | 3'-N-debenzoyl-2'-deoxytaxol N-benzoyltransferase |
|  | TraesCS2D02G568100 | 50S ribosomal protein L2 |  | TraesCS3B02G041100 | Acyl-CoA thioesterase, putative |
|  | TraesCS2D02G568200 | Agmatine coumaroyltransferase-2 |  | TraesCS3B02G041200 | Protein NEGATIVE REGULATOR OF RESISTANCE |
|  | TraesCS2D02G568300 | O-methyltransferase |  | TraesCS3B02G041300 | Disease resistance protein RPM1 |
|  | TraesCS2D02G568400 | DNA/RNA helicase protein |  | TraesCS3B02G041400 | Disease resistance protein (NBS-LRR class) family |
|  | TraesCS2D02G568500 | GATA transcription factor, putative |  | TraesCS3B02G041500 | F-box family protein |
|  | TraesCS2D02G568600 | GATA transcription factor, putative |  | TraesCS3B02G041600 | HIPL1 protein |
|  | TraesCS2D02G568700 | Disease resistance protein (NBS-LRR class) family | | TraesCS3B02G041700 | Alpha-glucosidase |
|  | TraesCS2D02G568800 | Disease resistance protein (NBS-LRR class) family | | TraesCS3B02G041800 | Translation initiation factor IF-2 |
|  | TraesCS2D02G568900 | Disease resistance protein |  | TraesCS3B02G041900 | Tryptophan synthase alpha chain |
|  | TraesCS2D02G569000 | F-box family protein |  | TraesCS3B02G042000 | Chromatin remodeling 8 |
|  | TraesCS2D02G569100 | Leucine-rich repeat receptor-like protein kinase family protein | | TraesCS3B02G042100 | F-box domain containing protein |
|  | TraesCS2D02G569200 | Ribosomal protein S13 |  | TraesCS3B02G042200 | F-box domain containing protein |
|  | TraesCS2D02G569300 | Cytochrome c oxidase subunit 3 |  | TraesCS3B02G042300 | GMP synthase, putative |
|  | TraesCS2D02G569400 | CC-NBS-LRR disease resistance protein |  | TraesCS3B02G042400 | AP2-EREBP transcription factor |
|  | TraesCS2D02G569500 | Eukaryotic aspartyl protease family protein, putative | | TraesCS3B02G042500 | P-loop containing nucleoside triphosphate hydrolases superfamily protein |
|  | TraesCS2D02G569600 | F-box family protein |  | TraesCS3B02G042600 | Signal peptidase subunit family protein |
|  | TraesCS2D02G569700 | Protein STAY-GREEN LIKE, chloroplastic |  | TraesCS3B02G042700 | Basic 7S globulin |
|  | TraesCS2D02G569800 | Pentatricopeptide repeat-containing family protein | | TraesCS3B02G042800 | Basic 7S globulin 2 |
|  | TraesCS2D02G569900 | S-adenosyl-L-methionine-dependent methyltransferases superfamily protein | | TraesCS3B02G042900 | Heat stress transcription factor A-9 |
|  | TraesCS2D02G570000 | Transmembrane protein, putative (DUF247) |  | TraesCS3B02G043000 | Translocator assembly/maintenance protein |
|  | TraesCS2D02G570100 | Transmembrane protein, putative (DUF247) |  | TraesCS3B02G043100 | Mitochondrial import inner membrane translocase subunit TIM22 |
|  | TraesCS2D02G570200 | F-box protein family |  | TraesCS3B02G043200 | Polyubiquitin |
|  | TraesCS2D02G570300 | RING/FYVE/PHD zinc finger superfamily protein | | TraesCS3B02G043300 | Leucine-rich repeat receptor-like protein kinase family protein |
|  | TraesCS2D02G570400 | RING/FYVE/PHD zinc finger superfamily protein | | TraesCS3B02G043400 | Receptor-like protein kinase |
|  | TraesCS2D02G570500 | Flowering time control protein FPA |  | TraesCS3B02G043600 | Protein kinase |
|  | TraesCS2D02G570600 | With no lysine (K) kinase 5 |  | TraesCS3B02G043700 | Protein kinase |
|  | TraesCS2D02G570700 | CsAtPR5 |  | TraesCS3B02G043800 | Leucine-rich repeat receptor-like protein kinase family protein |
|  | TraesCS2D02G570800 | CsAtPR5 |  | TraesCS3B02G043877 | NA |
|  | TraesCS2D02G570900 | BTB/POZ domain-containing protein TNFAIP1 isoform 1 | | TraesCS3B02G043900 | Cytochrome P450 |
|  | TraesCS2D02G571000 | Tubulin folding cofactor B |  | TraesCS3B02G044000 | Protein kinase |
|  | TraesCS2D02G571100 | Receptor kinase |  | TraesCS3B02G044100 | Protein kinase |
|  | TraesCS2D02G571200 | EamA-like transporter family protein |  | TraesCS3B02G044200 | Protein kinase |
|  | TraesCS2D02G571300 | 1-phosphatidylinositol-3-phosphate 5-kinase |  | TraesCS3B02G044300 | Protein kinase |
|  | TraesCS2D02G571400 | Chitinase |  | TraesCS3B02G044400 | Beta-galactosidase 8 |
|  | TraesCS2D02G571500 | DNA-directed RNA polymerase subunit |  | TraesCS3B02G044500 | Cullin-1 |
|  | TraesCS2D02G571600 | Chitinase |  | TraesCS3B02G044600 | Disease resistance protein RGA2 |
|  | TraesCS2D02G571700 | C2 calcium/lipid-binding and GRAM domain protein | | TraesCS3B02G044700 | Leucine-rich repeat receptor-like protein kinase family protein |
|  | TraesCS2D02G571800 | Histone H2A |  | TraesCS3B02G044800 | Leucine-rich repeat receptor-like protein kinase family protein |
|  | TraesCS2D02G571900 | RING/FYVE/PHD zinc finger protein |  | TraesCS3B02G044900 | Cytochrome P450 family protein, expressed |
|  | TraesCS2D02G572000 | Receptor-like protein kinase |  | TraesCS3B02G045000 | Leucine-rich repeat receptor-like protein kinase family protein |
|  | TraesCS2D02G572100 | Ataxia telangiectasia-mutated and RAD3-like protein | | TraesCS3B02G045100 | Leucine-rich repeat receptor-like protein kinase family protein |
|  | TraesCS2D02G572300 | 30S ribosomal protein S17 |  | TraesCS3B02G045200 | Leucine-rich repeat receptor-like protein kinase family protein |
|  | TraesCS2D02G572400 | F-box family protein |  | TraesCS3B02G045300 | Leucine-rich repeat receptor-like protein kinase family protein |
|  | TraesCS2D02G572500 | F-box protein, putative (DUF295) |  | TraesCS3B02G045400 | Aldo-keto reductase |
|  | TraesCS2D02G572600 | Protein kinase |  | TraesCS3B02G045500 | Calcium binding family protein |
|  | TraesCS2D02G572700 | Importin beta 1 |  | TraesCS3B02G045600 | Endo-1,4-beta-xylanase |
|  | TraesCS2D02G572800 | Photosystem II D2 protein |  | TraesCS3B02G045700 | Endo-1,4-beta-xylanase |
|  | TraesCS2D02G572900 | Photosystem II CP43 reaction center protein |  | TraesCS3B02G045800 | Phosphatidylinositol 4-phosphate 5-kinase 4 |
|  | TraesCS2D02G573000 | Photosystem II CP43 reaction center protein |  | TraesCS3B02G045900 | Calmodulin-binding protein-like |
|  | TraesCS2D02G573100 | Photosystem II reaction center protein Z |  | TraesCS3B02G046000 | Zinc finger, C2H2 |
|  | TraesCS2D02G573200 | Photosystem II reaction center protein M |  | TraesCS3B02G046100 | Delta(7)-sterol-C5(6)-desaturase |
|  | TraesCS2D02G573300 | Glutamyl-tRNA(Gln) amidotransferase subunit A | | TraesCS3B02G046200 | Ankyrin repeat family protein-like |
|  | TraesCS2D02G573400 | Chloroplast outer membrane translocon subunit, putative, expressed | | TraesCS3B02G046300 | F-box like protein |
|  | TraesCS2D02G573500 | CsAtPR5 |  | TraesCS3B02G046400 | F-box like protein |
|  | TraesCS2D02G573600 | Disease resistance protein (NBS-LRR class) family | | TraesCS3B02G046500 | Deoxyhypusine synthase |
|  | TraesCS2D02G573700 | Receptor-kinase, putative |  | TraesCS3B02G046600 | F-box family protein |
|  | TraesCS2D02G573800 | Disease resistance protein (NBS-LRR class) family | | TraesCS3B02G046700 | Receptor-like kinase |
|  | TraesCS2D02G573900 | Disease resistance protein (TIR-NBS-LRR class) | | TraesCS3B02G046800 | Eukaryotic translation initiation factor 4E |
|  | TraesCS2D02G574000 | Leucine-rich repeat receptor-like protein kinase family protein | | TraesCS3B02G046900 | Core-2/I-branching beta-1,6-N-acetylglucosaminyltransferase family protein, putative |
|  | TraesCS2D02G574100 | Protein kinase family protein |  | TraesCS3B02G047000 | Core-2/I-branching beta-1,6-N-acetylglucosaminyltransferase family protein, putative |
|  | TraesCS2D02G574200 | E3 ubiquitin-protein ligase |  | TraesCS3B02G047100 | Hydroxyacylglutathione hydrolase |
|  | TraesCS2D02G574300 | Disease resistance protein (TIR-NBS-LRR class) | | TraesCS3B02G047200 | GeBP-type transcription factor |
|  | TraesCS2D02G574400 | Disease resistance protein (NBS-LRR class) family | | TraesCS3B02G047300 | Hexose transporter |
|  | TraesCS2D02G574500 | Disease resistance protein (TIR-NBS-LRR class) family | | TraesCS3B02G047400 | Carboxyl-terminal peptidase (DUF239) |
|  | TraesCS2D02G574600 | Disease resistance protein (NBS-LRR class) family | | TraesCS3B02G047500 | RuvB-like helicase |
|  | TraesCS2D02G574700 | NBS-LRR-like resistance protein |  | TraesCS3B02G047600 | ABC transporter G family member |
|  | TraesCS2D02G574800 | Disease resistance protein (TIR-NBS-LRR class) family | | TraesCS3B02G047700 | Cotton fiber-like protein (DUF761) |
|  | TraesCS2D02G574900 | NBS-LRR resistance-like protein |  | TraesCS3B02G047800 | Hexose transporter |
|  | TraesCS2D02G575000 | Disease resistance protein (NBS-LRR class) family | | TraesCS3B02G047900 | Peptidyl-tRNA hydrolase family protein |
|  | TraesCS2D02G575100 | Cellulose synthase-like protein |  | TraesCS3B02G048000 | Zinc finger family protein |
|  | TraesCS2D02G575200 | Chaperone DnaK |  | TraesCS3B02G048100 | Mediator of RNA polymerase II transcription subunit 22 |
|  | TraesCS2D02G575300 | O-methyltransferase |  | TraesCS3B02G048200 | DUF1639 family protein |
|  | TraesCS2D02G575400 | O-methyltransferase |  | TraesCS3B02G048300 | Fanconi anemia group M protein |
|  | TraesCS2D02G575500 | DNA topoisomerase |  | TraesCS3B02G048400 | Membrane-associated kinase regulator |
|  | TraesCS2D02G575600 | Basic helix-loop-helix (bHLH) DNA-binding superfamily protein | | TraesCS3B02G048600 | Heat shock protein |
|  | TraesCS2D02G575700 | Alpha/Beta-Hydrolases superfamily protein |  | TraesCS3B02G048700 | Heat-shock protein |
|  | TraesCS2D02G575800 | 1-deoxy-D-xylulose 5-phosphate synthase |  | TraesCS3B02G048800 | Heat-shock protein |
|  | TraesCS2D02G575900 | Cellulose synthase-like protein |  | TraesCS3B02G048900 | Short-chain dehydrogenase/reductase |
|  | TraesCS2D02G576000 | Protein NRT1/ PTR FAMILY 5.5 |  | TraesCS3B02G049000 | DUF674 family protein |
|  | TraesCS2D02G576100 | Disease resistance protein RPM1 |  | TraesCS3B02G049100 | Receptor-like kinase |
|  | TraesCS2D02G576200 | NAC domain-containing protein, putative |  | TraesCS3B02G049800 | Heat shock protein |
|  | TraesCS2D02G576300 | NAC domain-containing protein, putative |  | TraesCS3B02G049900 | Heat shock protein |
|  | TraesCS2D02G576400 | NAC domain-containing protein, putative |  | TraesCS3B02G050000 | GDP-mannose 4,6-dehydratase |
|  | TraesCS2D02G576500 | NAC domain-containing protein, putative |  | TraesCS3B02G050100 | E3 ubiquitin-protein ligase |
|  | TraesCS2D02G576600 | Receptor kinase 1 |  | TraesCS3B02G050200 | E3 ubiquitin-protein ligase |
|  | TraesCS2D02G576700 | Rhomboid-like protein |  | TraesCS3B02G050300 | Glycosyltransferase |
|  | TraesCS2D02G576800 | DDB1-and CUL4-associated factor-like protein 1 | | TraesCS3B02G050400 | NB-ARC domain-containing disease resistance protein |
|  | TraesCS2D02G576900 | HXXXD-type acyl-transferase family protein |  | TraesCS3B02G050500 | Adp,atp carrier protein, mitochondrial |
|  | TraesCS2D02G577000 | Kinase family protein |  | TraesCS3B02G050600 | NB-ARC domain-containing disease resistance protein |
|  | TraesCS2D02G577100 | CsAtPR5 |  | TraesCS3B02G050700 | E3 ubiquitin-protein ligase |
|  | TraesCS2D02G577200 | E3 ubiquitin-protein ligase RNF126-A |  | TraesCS3B02G050800 | DNA-(apurinic or apyrimidinic site) lyase |
|  | TraesCS2D02G577300 | Disease resistance protein RPM1 |  | TraesCS3B02G050900 | E3 ubiquitin-protein ligase |
|  | TraesCS2D02G577400 | Disease resistance protein RPM1 |  | TraesCS3B02G051000 | Expansin |
|  | TraesCS2D02G577500 | Disease resistance protein RPM1 |  | TraesCS3B02G051100 | Expansin |
|  | TraesCS2D02G577700 | GEM-like protein 1 |  | TraesCS3B02G051200 | Expansin |
|  | TraesCS2D02G577800 | Auxin response factor |  | TraesCS3B02G051300 | Expansin |
|  | TraesCS2D02G577900 | UPF0503 protein, chloroplastic |  | TraesCS3B02G051400 | Expansin |
|  | TraesCS2D02G578000 | Carboxyl-terminal peptidase (DUF239) |  | TraesCS3B02G051500 | Expansin |
|  | TraesCS2D02G578100 | Nodulin homeobox |  | TraesCS3B02G051600 | Expansin |
|  | TraesCS2D02G578300 | Isoaspartyl peptidase/L-asparaginase |  | TraesCS3B02G051700 | B3 domain-containing protein Os01g0723500 |
|  | TraesCS2D02G578400 | GRF zinc finger family protein |  | TraesCS3B02G051800 | Expansin |
|  | TraesCS2D02G578500 | cDNA clone:J033115O13, full insert sequence | | TraesCS3B02G051900 | Expansin |
|  | TraesCS2D02G578600 | Tetratricopeptide repeat |  | TraesCS3B02G052000 | Expansin |
|  | TraesCS2D02G578700 | NADH-quinone oxidoreductase subunit F |  | TraesCS3B02G052100 | Lysostaphin resistance protein A |
|  | TraesCS2D02G578800 | Shaggy-like protein kinase 41 |  | TraesCS3B02G052200 | Heparan-alpha-glucosaminide N-acetyltransferase |
|  | TraesCS2D02G578900 | Zinc finger protein ZPR1 |  | TraesCS3B02G052300 | E3 ubiquitin-protein ligase |
|  | TraesCS2D02G579000 | 2-oxoglutarate (2OG) and Fe(II)-dependent oxygenase-like protein | | TraesCS3B02G052400 | E3 ubiquitin-protein ligase |
|  | TraesCS2D02G579100 | CsAtPR5 |  | TraesCS3B02G052500 | Lectin |
|  | TraesCS2D02G579200 | EamA-like transporter family protein |  | TraesCS3B02G052600 | Receptor-kinase, putative |
|  | TraesCS2D02G579300 | CsAtPR5 |  | TraesCS3B02G052700 | E3 ubiquitin-protein ligase |
|  | TraesCS2D02G579400 | 2-oxoglutarate (2OG) and Fe(II)-dependent oxygenase-like protein | | TraesCS3B02G052800 | E3 ubiquitin-protein ligase |
|  | TraesCS2D02G579500 | Glutathione S-transferase |  | TraesCS3B02G052900 | Glycosyltransferase |
|  | TraesCS2D02G579600 | Receptor-like protein kinase |  | TraesCS3B02G053000 | E3 ubiquitin-protein ligase |
|  | TraesCS2D02G579700 | Myb/SANT-like DNA-binding domain protein | | TraesCS3B02G053058 | NA |
|  | TraesCS2D02G579800 | Protein kinase family protein |  | TraesCS3B02G053100 | Lectin |
|  | TraesCS2D02G579900 | PLAC8 family protein |  | TraesCS3B02G053200 | Kinase family protein |
|  | TraesCS2D02G580000 | Formin-like protein |  | TraesCS3B02G053300 | E3 ubiquitin-protein ligase |
|  | TraesCS2D02G580100 | Transmembrane protein, putative (DUF594) |  | TraesCS3B02G053400 | E3 ubiquitin-protein ligase |
|  | TraesCS2D02G580200 | CsAtPR5 |  | TraesCS3B02G053500 | E3 ubiquitin-protein ligase |
|  | TraesCS2D02G580300 | CsAtPR5 |  | TraesCS3B02G053600 | Plant/Protein (Protein of unknown function, DUF538) |
|  | TraesCS2D02G580400 | C2H2-like zinc finger protein |  | TraesCS3B02G053700 | Protein kinase family protein |
|  | TraesCS2D02G580500 | Transmembrane protein, putative (DUF594) |  | TraesCS3B02G053900 | Phenylalanine ammonia-lyase |
|  | TraesCS2D02G580600 | CsAtPR5 |  | TraesCS3B02G054000 | E3 ubiquitin-protein ligase |
|  | TraesCS2D02G580700 | Ubiquitin |  | TraesCS3B02G054100 | Beta-fructofuranosidase, insoluble protein |
|  | TraesCS2D02G580800 | P-loop containing nucleoside triphosphate hydrolases superfamily protein | | TraesCS3B02G054200 | E3 ubiquitin-protein ligase |
|  | TraesCS2D02G580900 | CsAtPR5 |  | TraesCS3B02G054300 | E3 ubiquitin-protein ligase |
|  | TraesCS2D02G581000 | NADH-ubiquinone oxidoreductase chain 3 |  | TraesCS3B02G054400 | E3 ubiquitin-protein ligase |
|  | TraesCS2D02G581100 | 30S ribosomal protein S12 |  | TraesCS3B02G054500 | Cellulose synthase-like protein |
|  | TraesCS2D02G581200 | CoA ligase |  | TraesCS3B02G054700 | Receptor-like kinase |
|  | TraesCS2D02G581300 | RNA-binding region RNP-1 |  | TraesCS3B02G054800 | E3 ubiquitin-protein ligase |
|  | TraesCS2D02G581400 | Pathogenesis-related thaumatin-like protein |  | TraesCS3B02G054900 | E3 ubiquitin-protein ligase |
|  | TraesCS2D02G581500 | Agmatine coumaroyltransferase-2 |  | TraesCS3B02G055000 | E3 ubiquitin-protein ligase |
|  | TraesCS2D02G581600 | Ubiquitin carboxyl-terminal hydrolase 2 |  | TraesCS3B02G055100 | E3 ubiquitin-protein ligase |
|  | TraesCS2D02G581700 | Cytochrome P450 |  | TraesCS3B02G055200 | E3 ubiquitin-protein ligase |
|  | TraesCS2D02G581800 | GRF zinc finger family protein |  | TraesCS3B02G055300 | E3 ubiquitin-protein ligase |
|  | TraesCS2D02G581900 | CoA ligase |  | TraesCS3B02G055400 | Epoxide hydrolase 2 |
|  | TraesCS2D02G582000 | Agmatine coumaroyltransferase-2 |  | TraesCS3B02G055500 | GRF zinc finger family protein |
|  | TraesCS2D02G582100 | Transmembrane protein, putative (DUF594) |  | TraesCS3B02G055600 | Receptor-like kinase |
|  | TraesCS2D02G582200 | ATP synthase subunit alpha, chloroplastic |  | TraesCS3B02G055700 | Receptor-like kinase |
|  | TraesCS2D02G582300 | Kinase family protein |  | TraesCS3B02G055800 | Kinase, putative |
|  | TraesCS2D02G582400 | PI-PLC X domain-containing protein |  | TraesCS3B02G055835 | NA |
|  | TraesCS2D02G582500 | Kinase, putative |  | TraesCS3B02G055900 | Receptor-like kinase |
|  | TraesCS2D02G582600 | Kinase, putative |  | TraesCS3B02G056000 | Receptor-like protein kinase |
|  | TraesCS2D02G582700 | Kinase, putative |  | TraesCS3B02G056100 | Receptor-like kinase |
|  | TraesCS2D02G582800 | Kinase, putative |  | TraesCS3B02G056500 | Receptor-like kinase |
|  | TraesCS2D02G582900 | Peroxidase |  | TraesCS3B02G056600 | Protein kinase family protein |
|  | TraesCS2D02G583000 | Peroxidase |  | TraesCS3B02G056900 | Wall-associated kinase family protein |
|  | TraesCS2D02G583100 | Peroxidase |  | TraesCS3B02G057000 | Kinase-like protein |
|  | TraesCS2D02G583200 | Peroxidase |  | TraesCS3B02G057200 | Receptor-like protein kinase |
|  | TraesCS2D02G583300 | Major facilitator superfamily protein |  | TraesCS3B02G057300 | Receptor-like kinase |
|  | TraesCS2D02G583400 | Protein NRT1/ PTR FAMILY 5.5 |  | TraesCS3B02G057400 | Receptor-like kinase |
|  | TraesCS2D02G583500 | Protein NRT1/ PTR FAMILY 5.5 |  | TraesCS3B02G057500 | Receptor-like kinase |
|  | TraesCS2D02G583600 | Protein NRT1/ PTR FAMILY 5.5 |  | TraesCS3B02G057700 | Receptor-like kinase |
|  | TraesCS2D02G583700 | Peroxidase |  | TraesCS3B02G057800 | Wall-associated kinase |
|  | TraesCS2D02G583800 | Serpin-like protein |  | TraesCS3B02G057900 | Receptor-like kinase |
|  | TraesCS2D02G583900 | Peroxidase |  | TraesCS3B02G057947 | NA |
|  | TraesCS2D02G584000 | Peroxidase |  | TraesCS3B02G058000 | Receptor-like kinase |
|  | TraesCS2D02G584100 | Peroxidase |  | TraesCS3B02G058100 | Kinase-like protein |
|  | TraesCS2D02G584200 | F-box domain containing protein, expressed |  | TraesCS3B02G058300 | DNA topoisomerase |
|  | TraesCS2D02G584300 | Succinate dehydrogenase subunit 3 |  | TraesCS3B02G058400 | Receptor-like kinase |
|  | TraesCS2D02G584400 | ATP synthase epsilon chain |  | TraesCS3B02G058500 | Receptor-like protein kinase |
|  | TraesCS2D02G584500 | Disease resistance protein (TIR-NBS-LRR class) | | TraesCS3B02G058600 | Receptor-like kinase |
|  | TraesCS2D02G584600 | Peroxidase |  | TraesCS3B02G058700 | Kinase, putative |
|  | TraesCS2D02G584700 | Peroxidase |  | TraesCS3B02G059000 | Receptor-like kinase |
|  | TraesCS2D02G584800 | Peroxidase |  | TraesCS3B02G059017 | NA |
|  | TraesCS2D02G584900 | Tubby-like F-box protein |  | TraesCS3B02G059100 | Receptor-like kinase |
|  | TraesCS2D02G585000 | Plant/F1M20-13 protein |  | TraesCS3B02G059200 | Histone-lysine N-methyltransferase |
|  | TraesCS2D02G585100 | Prenylcysteine oxidase / farnesylcysteine lyase | | TraesCS3B02G059300 | GMP synthase [glutamine-hydrolyzing] |
|  | TraesCS2D02G585200 | Galactoside 2-alpha-L-fucosyltransferase |  | TraesCS3B02G059400 | Electron transport complex subunit D |
|  | TraesCS2D02G585300 | ABC transporter G family member |  | TraesCS3B02G059500 | GMP synthase [glutamine-hydrolyzing] |
|  | TraesCS2D02G585400 | B3 domain-containing protein |  | TraesCS3B02G059600 | D-Ala-D/L-Ala epimerase |
|  | TraesCS2D02G585500 | Receptor kinase-like protein |  | TraesCS3B02G059700 | D-Ala-D/L-Ala epimerase |
|  | TraesCS2D02G585700 | Disease resistance protein (NBS-LRR class) family | | TraesCS3B02G059800 | GRF zinc finger family protein |
|  | TraesCS2D02G585800 | Galactose oxidase/kelch repeat superfamily protein | | TraesCS3B02G059900 | Glycine-rich cell wall structural protein 2 |
|  | TraesCS2D02G585900 | PfkB-like carbohydrate kinase family protein |  | TraesCS3B02G060000 | RING/U-box superfamily protein |
|  | TraesCS2D02G586000 | F-box family protein |  | TraesCS3B02G060100 | GDSL esterase/lipase |
|  | TraesCS2D02G586100 | 30S ribosomal protein S11 |  | TraesCS6A02G115500 | S-adenosyl-L-methionine-dependent methyltransferases superfamily protein |
|  | TraesCS2D02G586200 | Thioredoxin |  | TraesCS6A02G115600 | Auxin-responsive family protein |
|  | TraesCS2D02G586300 | Cysteine proteinase inhibitor |  | TraesCS6A02G115700 | SAUR-like auxin-responsive protein family, putative |
|  | TraesCS2D02G586400 | tRNA uridine 5-carboxymethylaminomethyl modification enzyme MnmG | MQTL-6A-4 | TraesCS6A02G115800 | DNA/RNA-binding protein KIN17 |
|  | TraesCS2D02G586500 | WAT1-related protein |  | TraesCS6A02G115900 | Splicing factor 3B subunit 1 |
|  | TraesCS2D02G586600 | Cysteine protease, putative |  | TraesCS6A02G116000 | Dolichol kinase |
|  | TraesCS2D02G586700 | Cysteine proteinase inhibitor |  | TraesCS6A02G116100 | Unknown protein |
|  | TraesCS2D02G586800 | Cysteine proteinase inhibitor |  | TraesCS6A02G116200 | ATP-dependent RNA helicase |
|  | TraesCS2D02G586900 | Cysteine proteinase inhibitor |  | TraesCS6A02G116300 | Ribosomal protein L37, mitochondrial |
|  | TraesCS2D02G587000 | F-box family protein |  | TraesCS6A02G116400 | Glycosyltransferase |
|  | TraesCS2D02G587100 | Inorganic pyrophosphatase |  | TraesCS6A02G116500 | 26S proteasome non-ATPase regulatory subunit-like protein |
|  | TraesCS2D02G587200 | Ribulose bisphosphate carboxylase small chain, chloroplastic | | TraesCS6A02G116600 | Polynucleotide 5'-hydroxyl-kinase NOL9 |
|  | TraesCS2D02G587300 | Chaperone protein DnaJ |  | TraesCS6A02G116700 | Transmembrane protein |
|  | TraesCS2D02G587400 | LRR receptor-like kinase family protein |  | TraesCS6A02G116800 | Harpin-induced protein |
|  | TraesCS2D02G587500 | Lectin protein kinase family protein |  | TraesCS6A02G116900 | Harpin inducing protein |
|  | TraesCS2D02G587600 | Nonribosomal peptide synthetase 13 |  | TraesCS6A02G117000 | HLA class II histocompatibility antigen, DRB1-16 beta chain |
|  | TraesCS2D02G587700 | 60S ribosomal protein L28 |  | TraesCS6A02G117100 | DNA-binding protein BIN4 |
|  | TraesCS2D02G587800 | CsAtPR5 |  | TraesCS7B02G481700 | Multiple C2 and transmembrane domain-containing protein 1 |
|  | TraesCS2D02G587900 | rRNA N-glycosidase |  | TraesCS7B02G481800 | Chloroplastic group IIA intron splicing facilitator CRS1, chloroplastic |
|  | TraesCS2D02G588000 | Vacuolar iron transporter-like protein |  | TraesCS7B02G481900 | F-box family protein |
|  | TraesCS2D02G588100 | F-box family protein | MQTL-7B-5 | TraesCS7B02G482000 | Phytoene synthase |
|  | TraesCS2D02G588200 | Protein kinase, putative |  | TraesCS7B02G482100 | Protein argonaute |
|  | TraesCS2D02G588300 | CsAtPR5 |  | TraesCS7B02G482200 | Sucrose synthase |
|  | TraesCS2D02G588400 | UDP-N-acetylenolpyruvoylglucosamine reductase | | TraesCS7B02G482300 | NBS-LRR disease resistance protein |
|  | TraesCS2D02G588500 | Pentatricopeptide repeat-containing family protein | | TraesCS7B02G482400 | Chromodomain-helicase-DNA-binding family protein |
|  | TraesCS2D02G588600 | Kinase family protein |  | TraesCS7B02G482500 | Ribosomal RNA small subunit methyltransferase G |
|  | TraesCS2D02G588700 | Protein kinase, putative |  | TraesCS7B02G482600 | Putative beta-1,3-glucanase |
|  | TraesCS2D02G588762 | NA |  | TraesCS7B02G482700 | MADS-box transcription factor family protein |
|  | TraesCS2D02G588800 | CsAtPR5 |  | TraesCS7B02G482800 | NBS-LRR class disease resistance protein |
|  | TraesCS2D02G588900 | Pentatricopeptide repeat-containing family protein | |  |  |
